# RNA-seq analysis reveals alternative splicing under salt stress in cotton, *Gossypium davidsonii*

**DOI:** 10.1186/s12864-018-4449-8

**Published:** 2018-01-23

**Authors:** Guozhong Zhu, Weixi Li, Feng Zhang, Wangzhen Guo

**Affiliations:** State Key Laboratory of Crop Genetics & Germplasm Enhancement, Hybrid Cotton R & D Engineering Research Center, Ministry of Education, Nanjing Agricultural University, Nanjing, 210095 China

**Keywords:** RNA-seq, Alternative splicing, Splicing factor, Salt stress, *G. davidsonii*

## Abstract

**Background:**

Numerous studies have focused on the regulation of gene expression in response to salt stress at the transcriptional level; however, little is known about this process at the post-transcriptional level.

**Results:**

Using a diploid D genome wild salinity-tolerant cotton species, *Gossypium davidsonii*, we analyzed alternative splicing (AS) of genes related to salt stress by comparing high-throughput transcriptomes from salt-treated and well-watered roots and leaves. A total of 14,172 AS events were identified involving 6798 genes, of which intron retention (35.73%) was the most frequent, being detected in 3492 genes. Under salt stress, 1287 and 1228 differential alternative splicing (DAS) events were identified in roots and leaves, respectively. These DAS genes were associated with specific functional pathways, such as “responses to stress”, “metabolic process” and “RNA splicing”, implying that AS represents an important pathway of gene regulation in response to salt stress. Several salt response genes, such as pyrroline-5-carboxylate synthase (*P5CS*), K^+^ channel outward (*KCO1*), plasma membrane intrinsic protein (*PIP*) and *WRKY33* which were involved in osmotic balance, ion homeostasis, water transportation and transcriptional regulation, respectively, were identified with differential alternative splicing under salt stress. Moreover, we revealed that 13 genes encoding Ser/Arg-rich (SR) proteins related to AS regulation were differentially alternatively spliced under salt stress.

**Conclusion:**

This study first provide a comprehensive view of AS in *G. davidsonii*, and highlight novel insights into the potential roles of AS in plant responses to salt stress.

**Electronic supplementary material:**

The online version of this article (10.1186/s12864-018-4449-8) contains supplementary material, which is available to authorized users.

## Background

The removal of introns from immature mRNA by a process called “pre-mRNA splicing” occurs in the vast majority of eukaryotic protein-coding genes. In this process, particular exons of a gene may be included in or excluded from the final, processed messenger RNA (mRNA) from that gene. This process is known as alternative splicing (AS) [1]. AS is a ubiquitous mechanism in higher eukaryotes and contributes to both transcriptome and proteome diversity [2]. AS creates multiple mRNA transcripts from a single gene through the selection and utilization of alternative splice sites in the pre-mRNA via different splicing events, including exon skipping (ES), alternative donor site (AD), alternative acceptor site (AA), intron retention (IR) and other complicated forms of splicing [3]. The frequency of AS events varies significantly and some are gene- or species-specific. In animals, ES and IR are the most and least frequent, respectively. For example, approximately 35.2% of all AS events in humans are caused by ES, but only 0.01% by IR [4]. In contrast, IR is the most predominant form of AS in *Arabidopsis* [5], *Zea mays* [6], and *Gossypium raimondii* [7], whereas ES only accounts for a small proportion.

AS is accomplished by spliceosomes, which are high molecular weight complexes that are assembled at every intron [8, 9]. They contain five small nuclear ribonucleoprotein particles (snRNPs) and over 200 additional proteins. The identification of splice sites under particular cellular conditions is related to the interaction of additional proteins, globally designated as splicing factors (SFs), that guide spliceosomal components and thereby the spliceosome to the respective splice sites [10]. The main families of these SFs are the Ser/Arg-rich (SR) proteins and heterogeneous nuclear ribonucleoprotein particle (hnRNP) proteins. These proteins bind specific sequences in the pre-mRNA called intronic or exonic splicing enhancer or suppressor sequences [11]. Splice site selection reflects the relative occupation of these sequences and interactions between different proteins on a pre-mRNA molecule. In both animals and plants, many SFs/RNA binding proteins (RBPs) and some core spliceosomal components themselves undergo AS in response to signals, and control their own levels and those of other SFs via AS [12, 13]. In addition, the activity of SFs can be regulated by posttranslational modification in response to environmental cues [14].

AS is involved in many physiological processes, as well as responses to biotic and abiotic stresses in plants [12, 15, 16]. Since the significance of AS from SR protein splicing factor was reported [17], many studies had focused on how AS influences important developmental and signaling pathways. It has been demonstrated that ultraviolet (UV) irradiation can induce cell apoptosis by affecting the expression of apoptotic genes in an AS-dependent way [18]. Splicing variants of ABI3 were influenced by a plant splicing factor (SUA) in seed germination, implying that AS participates in both ABA signaling and response to abiotic stresses [19]. Transcriptomic analysis accelerates the identification of new splicing junctions [20]. Using RNA-seq data, genome-wide AS analysis has been conducted in several plant species, such as *Oryza sativa* [21], *Zea mays* [6], *Glycine max* [22] and *Arabidopsis* [5]. The potential roles of AS in the response to salt stress in *Arabidopsis* [23] and to heat stress in *Physcomitrella patens* have been further elucidated [24]. In addition, some splice variants of SR proteins, which are important splicing regulators, were identified in *Arabidopsis* under high- and low-temperature stress, and they might, in turn, alter the splicing of other pre-mRNAs [12, 25]. These findings demonstrated that AS is influenced by abiotic stress and, in turn, AS also plays a role in regulating gene expression.

Salinity is one of the most brutal environmental stresses that can hamper crop productivity worldwide. Although cotton is a relatively salt-tolerant species, its growth and development can still be greatly affected by adverse salt conditions [26]. Based on the analysis of the evolution and domestication of cotton using the sequenced *G. hirsutum* acc. TM-1 genome, it has been implied that the D-subgenome of tetraploid cotton species is a major donor for stress tolerance [7]. Although the mechanism of AS in *G. raimondii* has been investigated, little is known about AS in response to salt stress in cotton. In the previous study, using transcriptomes comparison from salt-treated and well-watered roots and leaves of *G. davidsonii*, a diploid D-genome wild salinity-tolerant cotton species, we detected that salt overly sensitive (SOS) and reactive oxygen species (ROS) signaling pathways were involved in salt stress tolerance, and photosynthesis pathways and metabolism played important roles in ion homeostasis and oxidation balance. We also found that alternative splicing is induced by salt stress in cotton [26]. However, deeper analysis between AS and salt stress remains to be investigated. Here, based on the high-throughput transcriptomes from salt-treated and well-watered roots and leaves in *G. davidsonii*, we systematically investigated the global dynamics of AS and elucidated the relationship between AS and salt stress in cotton. We found that the number of AS events under stress conditions was higher than that under normal conditions, indicating that AS plays important roles in the response to salt stress. We also detected SRs involved in AS regulation and investigated their differential expression and AS under salt stress. This study not only provides a comprehensive view of AS in cotton, but also highlights novel insights into the potential roles of AS in plant responses to salt stress.

## Results

### Prediction of gene isoforms

**Fig. 1:**
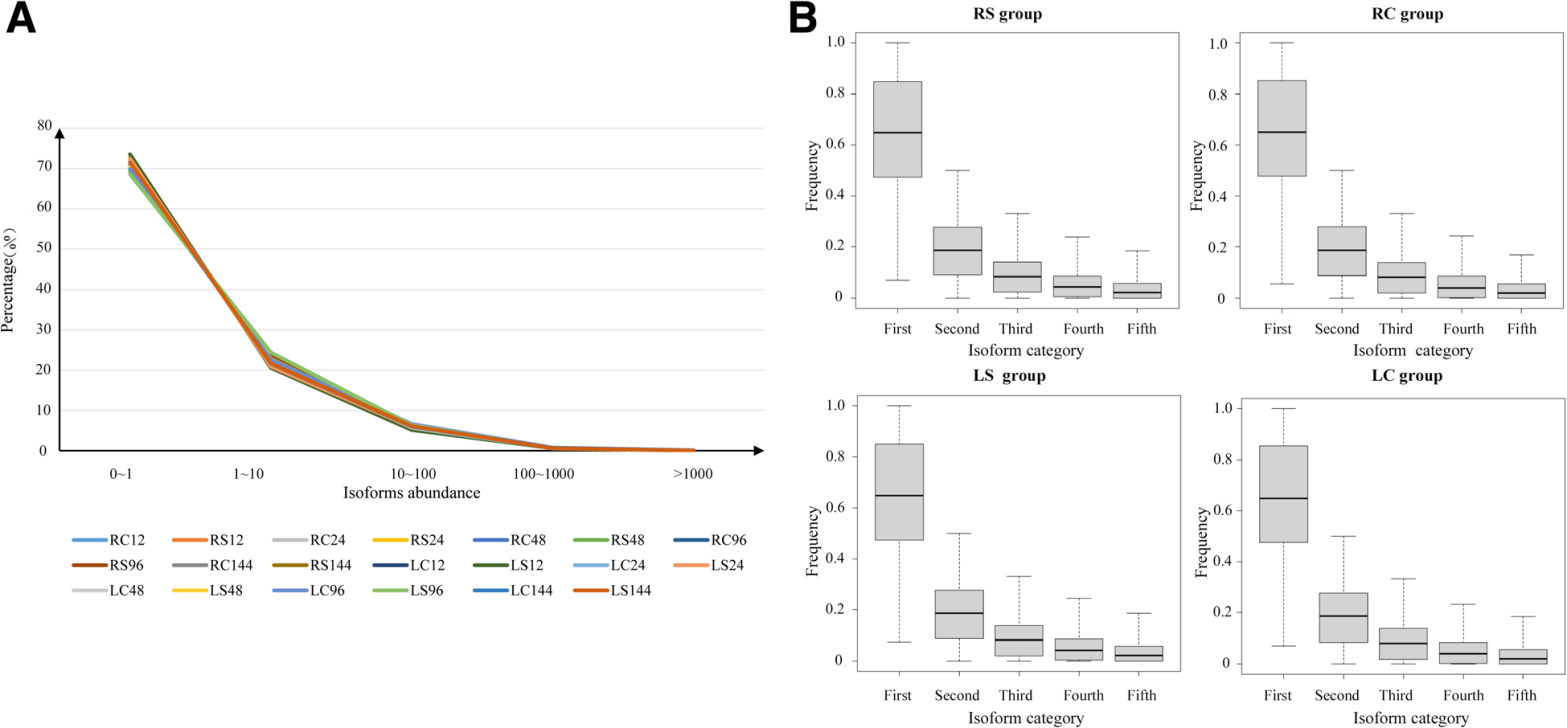
Isoforms abundance distribution in *G. davidsonii*. **a** The isoforms abundance distribution across 20 samples with two biological replicates. The isoforms abundance is evaluated with FPKM. **b** Frequency of the top five most abundance isoforms in each gene across four groups. RC: the roots of well-watered control plants, RS: the roots of salt-stressed plants, LC: the leaves of well-watered controls plants, LS: the leaves of salt-stressed plants. The subsequent number represents the time point post treatment

It is well known that multiple mRNA transcripts, also called gene isoforms, can be generated from a single gene via splicing events. Using RNA-seq data (Accessions: SRP061663), which were collected from both roots and leaves at 12, 24, 48, 96, and 144 h post salt stress (200 mM NaCl) in *G. davidsonii*, with data in normal conditions as controls [26], the splicing events involved in novel exons and novel intergenic transcripts were identified by mapping these data to the sequenced *G. raimondii* genome. The RNA-seq assays revealed 202,762 isoforms and 47,698 unigenes in total 40 libraries. Nevertheless, expression profiles revealed most isoforms were low abundance, with 70% lower than one fragments per kilobase per million reads (FPKM), about 20% between 1 and 10 FPKM, and less than 10% is over 10 FPKM (Fig. 1a). Expression level below 1 FPKM is thought to be beyond the limit of protein detection [27–29]. It means more than 70 percentage isoforms predicted in our data will not be translated into function proteins. This explains why the number of detected proteins is much less than the number of predicted isoforms. Meanwhile, it also means the low abundance isoforms cannot represent the function of multi-isoforms genes. In order to investigate the proportion of each isoform in corresponding gene, we grouped 40 libraries into four groups, the roots of well-watered control plants (RC), the roots of salt-stressed plants (RS), the leaves of well-watered controls plants (LC) and the leaves of salt-stressed plants (LS), and performed the statistics of top five most abundant isoforms for multi-isoforms genes (Fig. 1b and Additional file 1: Figure S1). Top1 isoforms in each group occupied greater 60% gene abundance and top two occupied less than 30%. The remaining isoforms covered very small part in gene abundance. This result indicated that the genes with top one or top two abundance isoforms were major contributors for multi-isoforms. In order to reduce the false positive of computer predicting and effectively mine the functional isoforms, the expression threshold value of isoform was detected using the empirical method [30]. We identified 2.6 FPKM for each isoform with repeatedly detected in each biological replicate for further analysis (Additional file 2: Figure S2). Following the criteria, 58,909 isoforms with 29,368 high confidence unigenes were identified. Of the predicted genes, 46.13% (13,546) had two or more isoforms and 53.87% (15,822) had only one isoform. In addition, 81.51% of the isoforms (48,019) were involved in 21,527 unigenes with two or more exons and 18.49% (10,890) had only one exon (Additional file 3: Figure S3). These multi-isoforms genes or multi-exons isoforms have the potential to generate AS events. In addition, 40,016 isoforms were detected in RC group, 41,443 in RS group, 39,758 in LC group, and 40,572 in LS group. We found that the number of isoforms increased in both roots (3.57%) and leaves (2.05%) under salt stress (Table 1).

**Table 1:** Increased isoforms under salt stress in roots and leaves, respectively

Treatment	Isoform no.	Gene no.	Isoform per gene
RC	40,016	22,941	1.74
RS	41,443	23,658	1.75
LC	39,758	22,993	1.73
LS	40,572	23,294	1.74
Total	58,909	29,368	2.01

*RC* root well-watered control, *RS* root salt-stressed treatment, *LC* leaf well-watered control, *LS* leaf salt-stressed treatment

### Identification of alternative splicing events

**Fig. 2:**
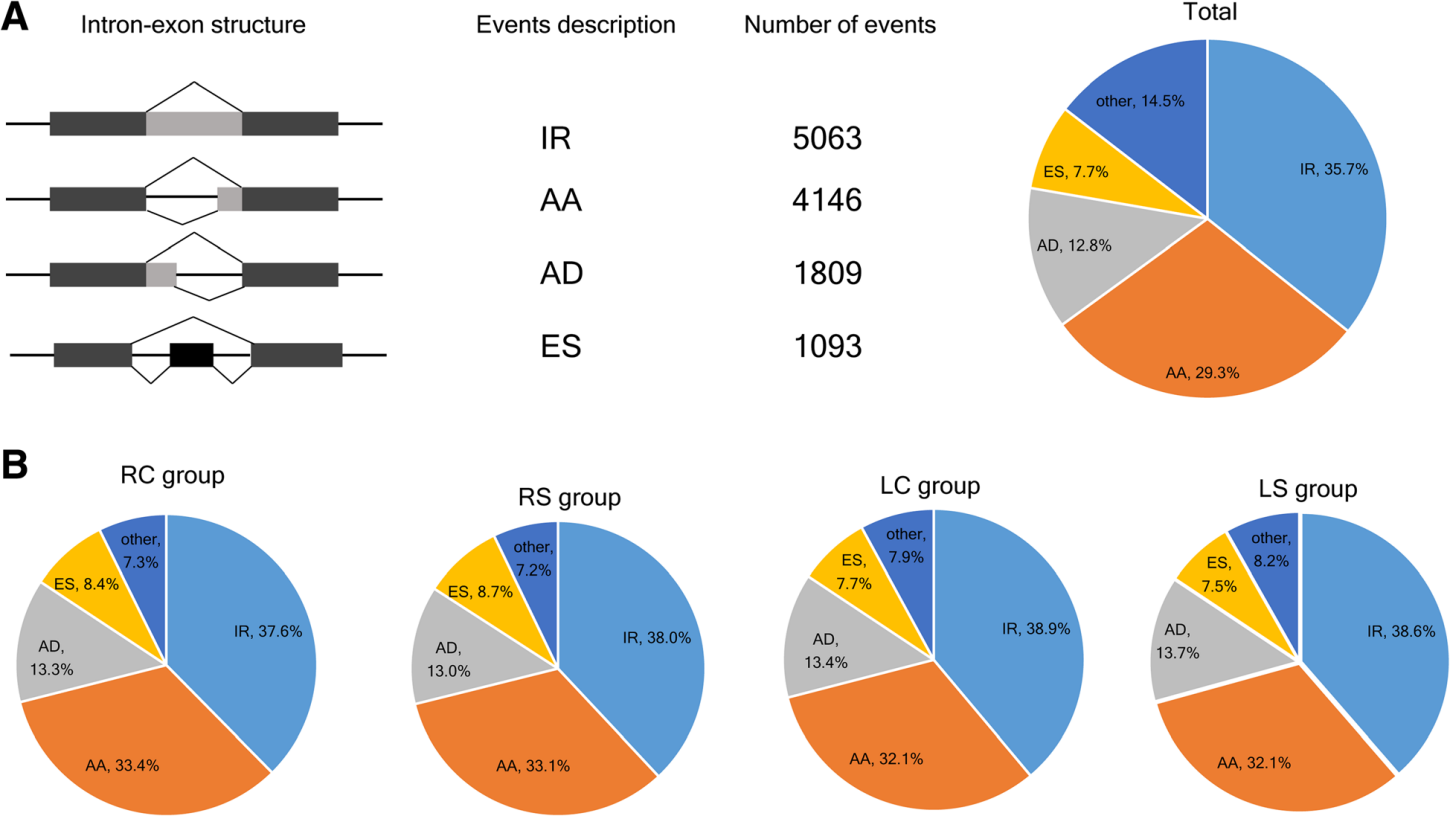
Distribution of alternative splicing events. **a** Four basic AS events in all 40 libraries. The first column illustrates the intron-exon structure of the AS events, followed by its description, the number of events and percentage. **b** The distribution of AS events in RC, RS, LC and LS group, respectively. The pie chart represents the frequency of each AS event

**Fig. 3:**
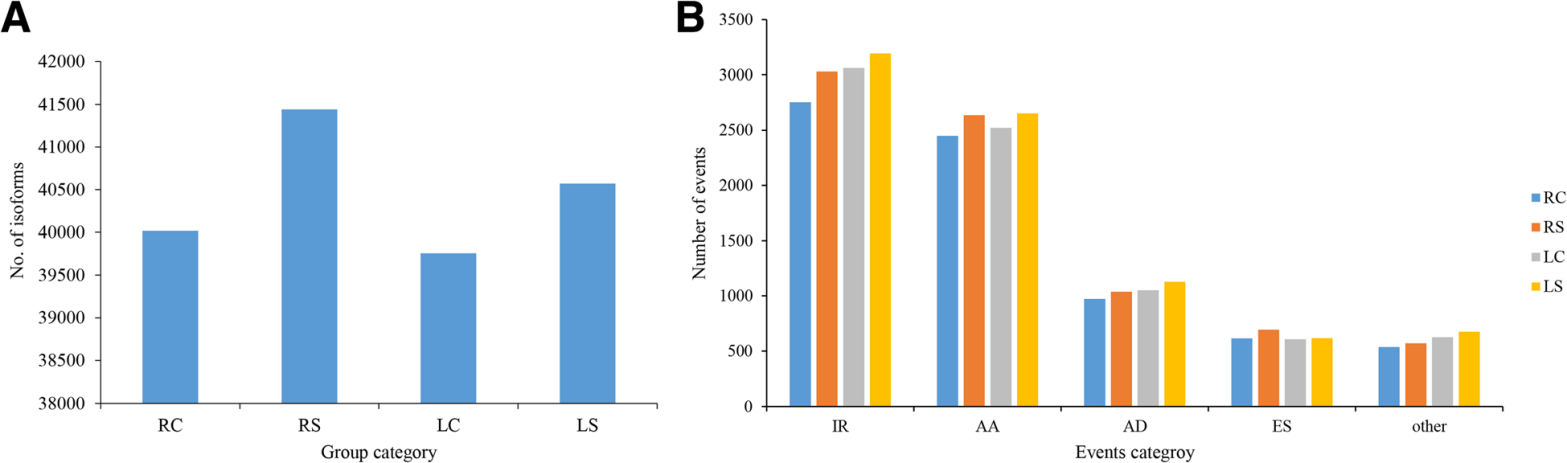
Number of isoform and AS events in four groups. **a** The number of isoforms in four groups. **b** The number of AS events in four groups

**Fig. 4:**
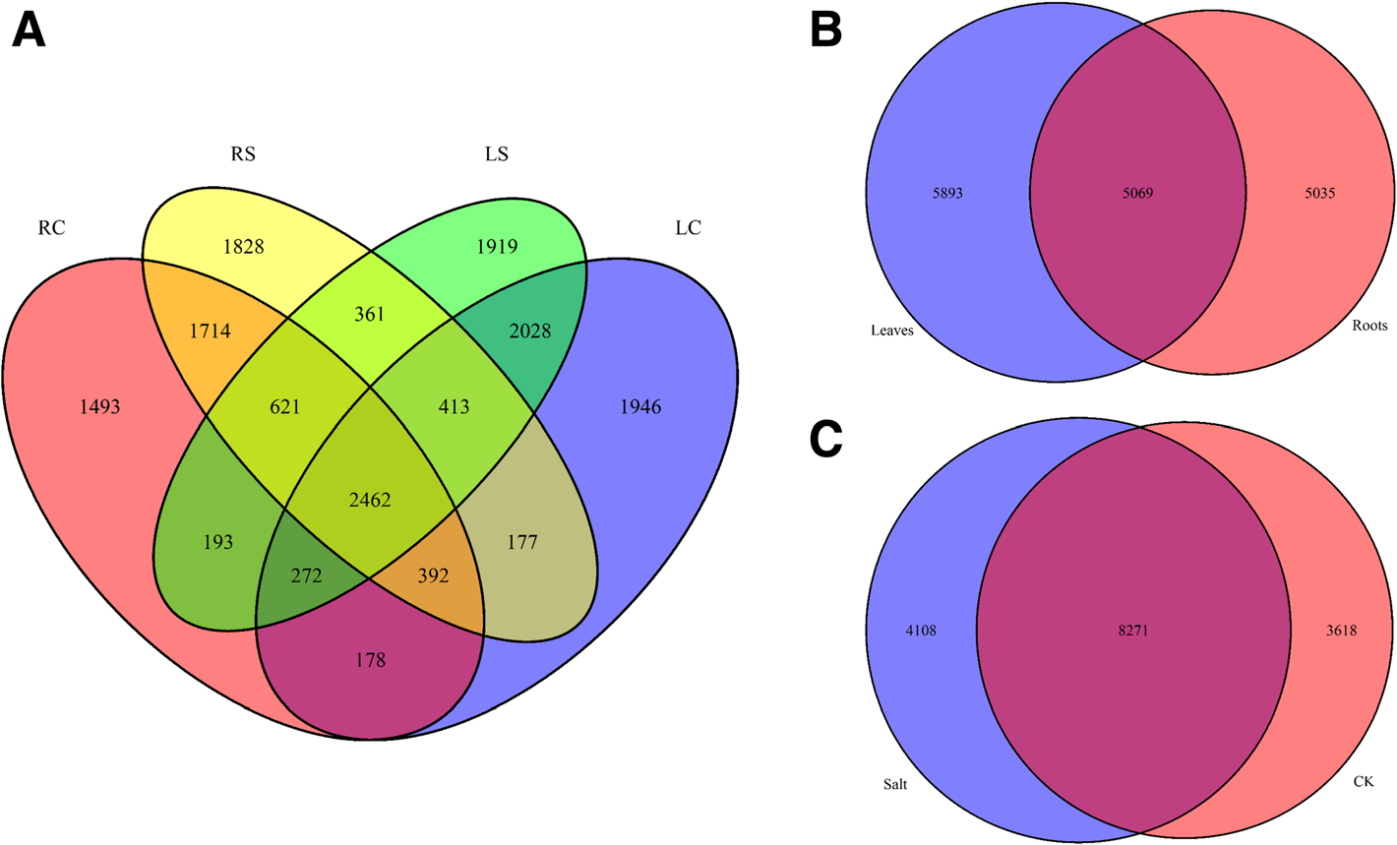
Venn diagram of alternative splicing events in roots and leaves at normal and salt stress condition. **a** Venn diagram of the overlap of alternative splicing events in four groups. **b** The common and specific alternative splicing events between roots and leaves. **c** The common and specific alternative splicing events between well-watered and salt stress condition

We used ASTALAVISTA to identify and classify the different types of alternative splicing. A total of 14,172 AS events were identified from 6798 genes in all 40 libraries, implying that nearly 31.58% of multi-exonic genes (6798/21,527) in roots or leaves underwent alternative splicing in *G. davidsonii*. We found that four basic AS types accounted for 85% of all AS events (Fig. 2a and Table 2). IR and ES events were the most and least frequent, and accounted for 35.73% (5063) and 7.71% (1093) of cases, respectively. AA events (29.25%) were more frequent than AD events (12.76%). These results are consistent with those of previous reports [5, 16, 31], which identified IR as the most frequent type of event in plant. Further, the distribution of four basic alternative splicing events in tissue- or treatment-specific groups were consistent with total events described above (Fig. 2b and Additional file 4: Table S1). As the isoforms increased under salt stress condition (Fig. 3a), all four basic events were increased in both roots and leaves post salt stress (Fig. 3b and Additional file 4: Table S1). It is consistent with the previous studies that AS was induced by abiotic stress [23, 32]. Only 2462 common events (17% of total events) in four groups were detected, which implied that most events were tissue or salt stress specific (Fig. 4a). In detail, 5069 AS events were present in both leaf and root tissues, while 5035 and 5893 events were root- or leaf-specific (Fig. 4b). Eight thousand two hundred seventy-one AS events were present in tissues under both stress and control conditions, while 3618 and 4108 events were specific to normal and salt stress conditions, respectively (Fig. 4c). Therefore, AS events in *G. davidsonii* differed more between tissues than under stress treatment (*p* < 0.001, Fisher’s exact tests).

**Table 2:** Number of AS events in total libraries

Events^a^	Events no.	Events rate	Gene no.	Gene rate^b^	AS per gene
AA	4146	29.25%	3065	45.08%	1.35
AD	1809	12.76%	1526	22.45%	1.19
ES	1093	7.71%	888	13.06%	1.23
IR	5063	35.73%	3492	51.37%	1.45
Other	2061	14.54%	1171	17.23%	1.76
Total	14,172	100%	6798	149.19%	2.08

^a^*AA* alternative acceptor site, *AD* alternative donor site, *ES* exon skipping, *IR* intron retention

^b^The total gene rate is higher than 100% is due to one gene may undergo two or more AS events

### Characteristics of different AS types

**Fig. 5:**
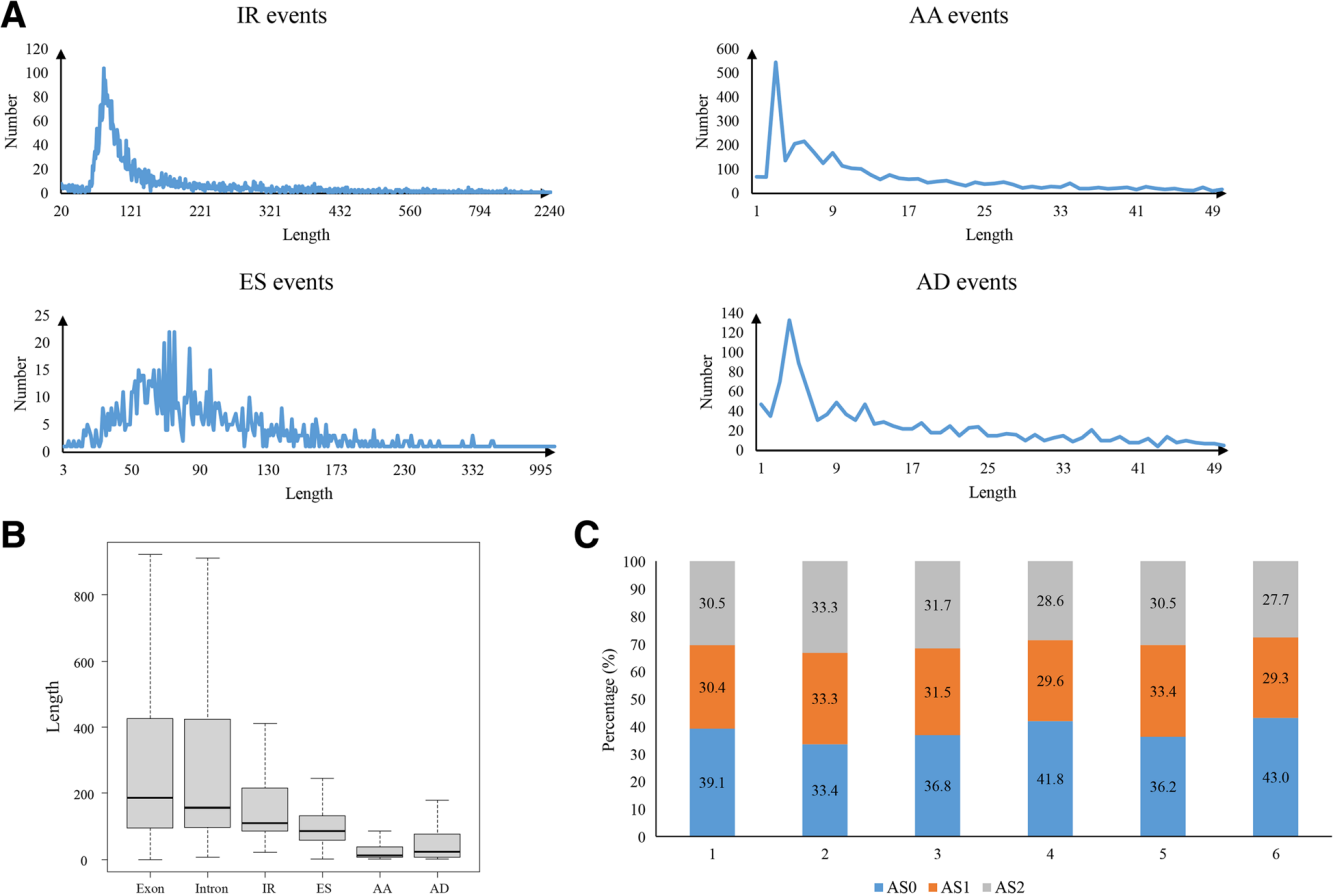
Length size of four basic alternative splicing events. **a** The length distribution of four basic alternative splicing events. To AA and AD events, the length more than 50 bp is few and not be shown in the chart. **b** length difference among exon in whole genome, intron in whole genome and four basic alternative splicing events. **c** Distribution in AS_0_, AS_1_, and AS_2_ types and their frequency, respectively

We found that the event length of the different AS types showed divergence (Fig. 5a). For IR, the retained intron length ranged from 21 to 2771 bp, with the largest number of events at 83 bp, which was similar to soybean [22] and shorter than rice [33]. Our analysis also showed that the most frequent AA length was 3 bp, and the most frequent AD length was 4 bp, which is in agreement with previous findings from other species [22, 34, 35]. The skipping length in ES has multi-peaks, ranging from 20 events at ~ 69 bp, 22 at ~ 72 bp, 22 at ~ 75 bp, and 19 at ~ 84 bp. We also found both length of intron in IR events and exon in ES events were significantly (*p* < 0.01, Student’s *t* test) shorter than the average length of intron and exon in total genome (Fig. 5b). In addition, according to the remainder when the length of events is divided by 3, we group each event into three categories: splicing events with length of multiples of three nucleotides named as AS_0_, the remainder 1 as AS_1_ and the remainder 2 as AS_2_. In general, splicing of AS_1_ or AS_2_ changes the reading frame, which either alters protein C termini or introduces premature termination codons (PTC) downstream from the splice junction, but splicing of AS_0_ does not. In total, AS_0_ type was dominant in four AS events and exon in total genome, except the intron (Fig. 5c). Especially, 41.8% in ES and 43.0% in AA were AS_0_ type, higher than AS_1_ and AS_2_ types, and the four peaks of ES length were all AS_0_ type. Even though each of three types in the intron accounted for 33.3% of total genome in average, all AS_0_ type of intron in IR events was 36.8% higher than the other two type which accounted for 31.5 and 31.7%, respectively. The result implies that more AS events in cotton undergo evolutionary pressure to preserve the reading frame.

### Changes in splicing patterns associated with stress response

**Fig. 6:**
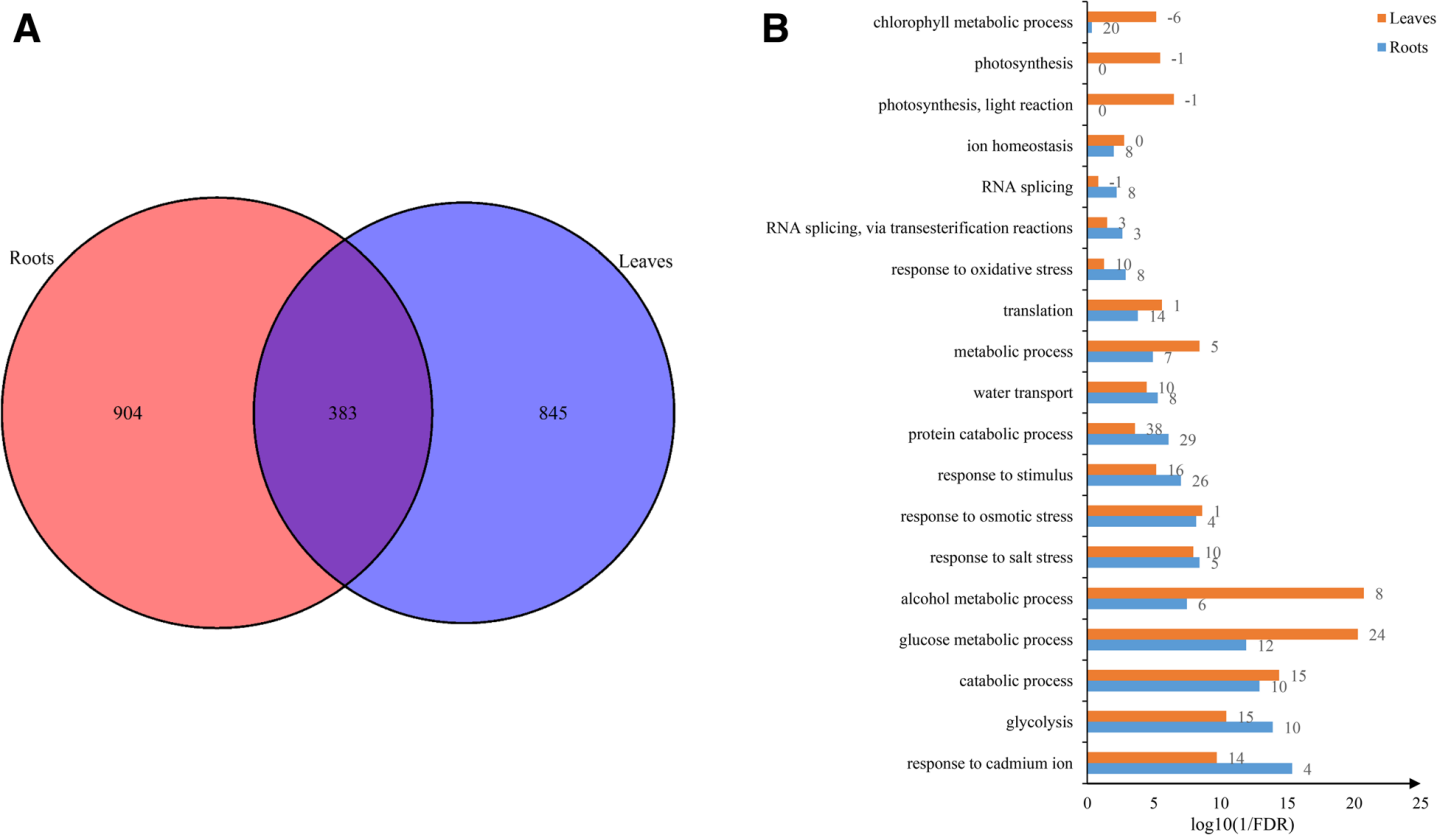
DAS and functional categorization of the related genes in roots and leaves under salt stress. **a** The common and specific DAS genes between roots and leaves. **b** Functional categorization with biological process of DAS genes in roots and leaves under salt stress. The positive numbers indicate the increased isoforms and the negative numbers indicate the decreased isoforms under salt condition

To investigate the potential influence of salt-stress-induced AS on cellular processes, we analyzed functional categories related to genes with AS. Totally, we identified 1287 and 1228 differential alternative splicing events (DAS), involved in 808 genes in roots and 791 genes in leaves, under salt stress (Fig. 6a). The length distribution of these DAS events was generally consistent with the AS events described above (Additional file 5: Figure S4). And AS_0_ type was also dominant in four AS events, even occupied 84% of total DAS events in ES type (Additional file 6: Figure S5). To determine whether the DAS events were affected by gene transcription, we compared the DAS genes with the differential expressed genes identified in our previous study [26]. Only a small subset of DAS genes (18.02% in roots and 21.63% in leaves) overlapped with the differential expressed genes (Additional file 7: Figure S6). This result suggests that the transcriptional activity of genes induced by salt stress has no significant effect on DAS events, with the similar report in *Physcomitrella patens* [24].

Based on the Gene Ontology (GO) analysis, we found that the differential alternative splicing overrepresentation of different GO categories in both roots and leaves, and the number of isoforms involved in related terms increased under salt stress (Fig. 6b and Additional file 8: Table S2). Most terms were common enriched in roots and leaves, such as “response to salt stress”, “water transport” and “alcohol catabolic process”. In addition, photosynthesis related terms in leaves, such as “photosynthesis” and “chlorophyll metabolic process”, were exclusively overrepresented. Although most biological process were commonly overrepresented in roots and leaves, tissue-specific regulation of DAS was distinct, and less than 20% DAS genes were simultaneously detected in the two tissues (Fig. 6a).

**Fig. 7:**
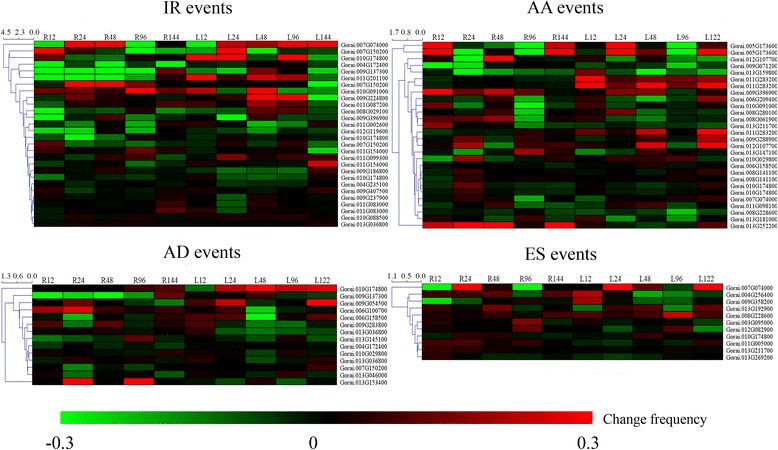
Splicing pattern of DAS genes related to salt stress response. R: Change frequency under salt stress in roots. L: Change frequency under salt stress in leaves. The subsequent number represent the time point post salt stress. Change frequency indicates the percentage of AS events increase or decrease post salt stress: Change frequency = (FPKM (AS events isoforms)/FPKM (total isoforms))_salt_ – (FPKM(AS events isoforms)/FPKM(total isoforms))_CK_. Red represents the increased and green represents the decreased AS events under salt stress

We found 81 DAS events involved in 58 genes in “response to salt stress” terms (Additional file 9: Table S3). Most of them were regulated by IR and AA events. The expression of AS events was various under salt stress, with some increased and the other decreased (Fig. 7). We also performed the correlation analysis between AS frequency change values and fold change values of gene expression for salt response genes (Additional file 10: Table S4). The results showed that only 5% (4/81) DAS events were significantly correlated with their gene expression, implying that AS acts an independent regulatory pattern comparing to transcription regulation under salt stress. In the DAS genes, we found that some important genes involved in the response to salt stress, such as Pyrroline-5-carboxylate synthase (*P5CS*, *Gorai.012G107700*), K^+^ channel outward (*KCO1*, *Gorai.013G153400*) and plasma membrane intrinsic protein (*PIP*, *Gorai.011G098100*), generated novel AS isoforms. *P5CS* plays crucial roles in the proline biosynthesis pathway [36] and proline is an important compatible solute for osmosis homeostasis under salt stress [37]. *KCO1* is involved in the K^+^ transport, which contributes to ion homeostasis [38]. *PIP* is a member of the aquaporin family, which is a crucial water channel protein involved in the salt stress response [39]. In addition, we also found some transcription factors that were related to the salt stress response, such as *WRKY* (*Gorai.012G119600*) [40], *MYB* (*Gorai.009G288900*) [41] and *bHLH* (*Gorai.009G396900*) [42]. Taken together, there exists the complex regulating mechanism in response on salt stress in cotton.

### Splicing factors involved in AS under salt stress

RNA splicing genes, especially those encoding splicing regulators, are known to be actively alternatively spliced under stress conditions [43]. The serine/arginine-rich (SR) proteins are known to be involved in pre-mRNA splicing processes and regulate alternative splicing by changing the splice site selection in a concentration dependent-manner [44]. In this study, 29 SR orthologous genes (Additional file 11: Table S5) were identified in the *G. raimondii* annotation information (http://genome.jgi.doe.gov/). Of them, 13 (12 in roots and seven in leaves) were differentially alternatively spliced under salt stress (Table 3). However, only two SR orthologous genes (*Gorai.009G393200*, *Gorai.010G201600*) were differential expression (up-regulated) under salt stress [26], indicating that SR genes were more inclined to be regulated by post-transcriptional level instead of transcriptional level. Following the *Arabidopsis* SR protein family divided into six subfamilies [25], we found that most differential splicing SR proteins in cotton belonged to RSZ subfamily, which was not reported in *Arabidopsis*, implying the different splicing regulation under salt stress between in cotton and *Arabidopsis*.

**Table 3:** The components of splicing machinery involved in regulation of salt stress responses in *G. davidsonii*

ID in *G. raimondii*	ID in *A. thaliana*	Gene symbol	RDAS	LDAS
Gorai.010G203100	AT3G53500	RSZ32	AA	AA
Gorai.011G242700	AT3G53500	RSZ32	AA	
Gorai.013G024100	AT3G53500	RSZ32	AA	AA
Gorai.004G044100	AT3G61860	RSP31	AA	
Gorai.002G165700	AT4G31580	SRZ22	IR	AA
Gorai.010G121100	AT4G31580	SRZ22	ES	AA
Gorai.010G245400	AT4G31580	SRZ22	IR	IR
Gorai.005G220000	AT5G64200	SC35	IR	IR
Gorai.007G128300	AT1G02840	SRP34	other	
Gorai.002G252500	AT2G37340	RSZ33	IR	
Gorai.012G091800	AT2G37340	RSZ33	AA	
Gorai.010G126300	AT3G13570	SCL30A		ES
Gorai.008G280300	AT4G25500	ATRSP40, RSP35	AD	

*RDAS* differential alternative splicing types in roots, *LDAS* differential alternative splicing types in leaves

### Validation of predicted AS and DAS events

**Fig. 8:**
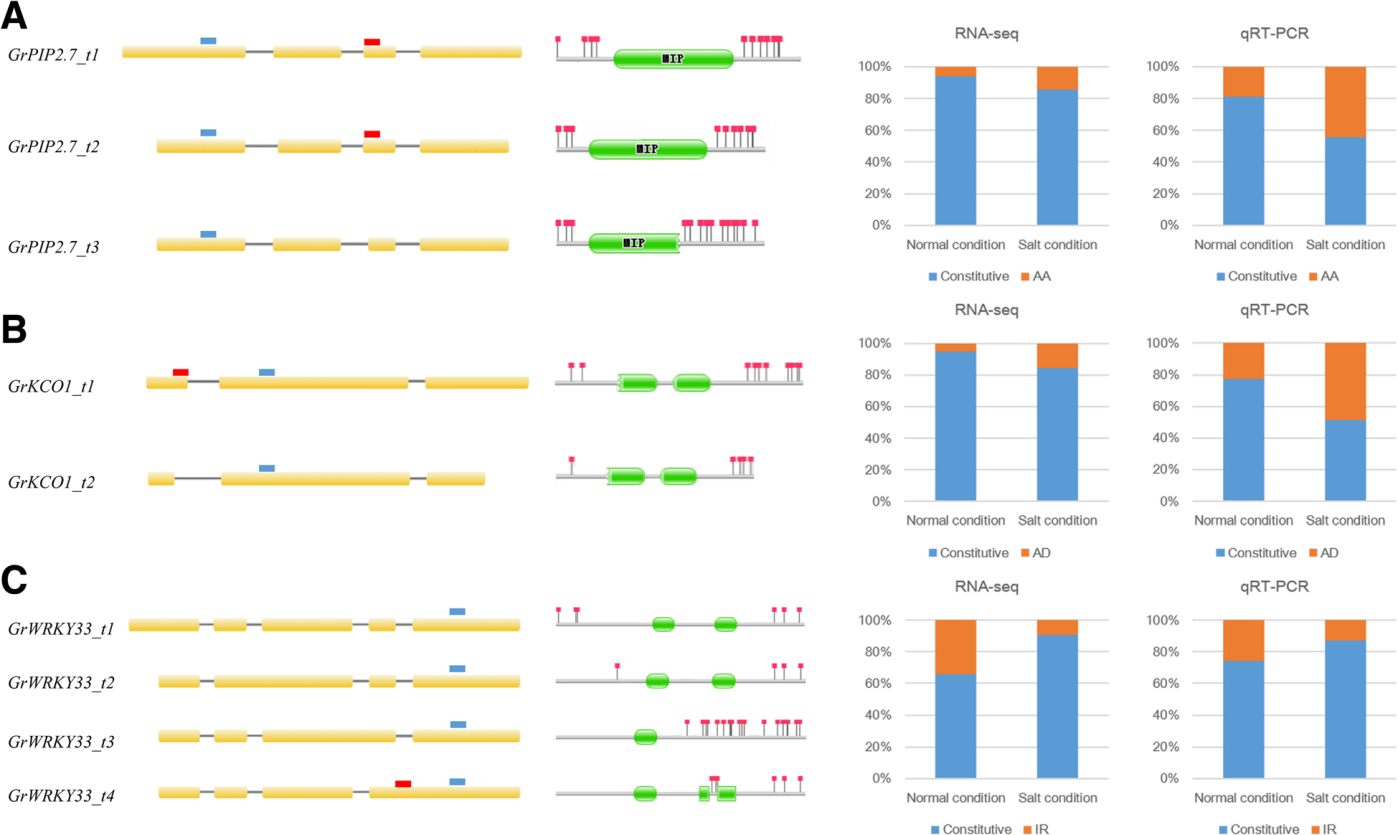
Validation on DAS events involved in salt stress by qRT-PCR. The first column showed the exon structure of each gene isoform. The red rectangle represents the PCR product at AS events loci, and the blue rectangle represents the PCR product at the constitutive exon loci. The second column showed the corresponding prediction of termination codon and functional domain. The third column showed the AS events ratio in RNA-seq data. The fourth column showed the AS events ratio in qRT-PCR analysis. **a** *GrPIP2.7*; **b** *GrKCO1*; **c** *GrWRKY33* orthologs in *G. davidsonii*, respectively

In order to evaluate the reliability of computationally predicted AS events, nine AS events with the length ranging from 60 to 200 bp for detection by agarose gel analysis, were randomly selected and validated by RT-PCR using intron-flanking primers (Additional file 12: Figure S7 and Additional file 13: Table S6). As a result, the PCR products were more than one in agarose gel corresponding to the AS events. Meanwhile, we also found the amplification products difference in different tissues or treatments, implying that AS was complicated and sensitive in different conditions. Further, ten DAS genes, which included three salt stress response genes reported previously and seven other induced genes, were validated by evaluating the relative abundance of the splicing event by qRT-PCR (Fig. 8, Additional file 14: Table S7 and Additional file 15: Figure S8), and the ratio between normal condition and salt condition for each variant. In total, 80% (8/10) predicted events were able to be detected by qRT-PCR. The events that were unable to be validated may be the result of false computational predictions or low transcript expression. A plasma membrane intrinsic protein (*GrPIP2.7*, *Gorai.011G098100*) had three isoforms. *GrPIP2.7_t1* was the dominant isoform that highly expressed in both roots and leaves, followed by *GrPIP2.7_t2* and *GrPIP2.7_t3*. *GrPIP2.7_t3* was generated by an AS event found at an alternative 3′ acceptor site (AA) which introduced a PTC at the MIP domain on the third exon. RNA-seq data and qRT-PCR revealed that the proportion of T3 increased under salt stress (Fig. 8a). K^+^ channel outward (*GrKCO1*, *Gorai.013G153400*) had two isoforms. *GrKCO1_t1* was the dominant isoform and an alternative 5′ donor site (AD) located on the first exon within 5′ UTR region that would not change the integrity of CDS region. RNA-seq data and qPCR revealed that the proportion of *GrKCO1_t* 2 increased under salt condition (Fig. 8b). A WRKY transcription factor (*GrWRKY33*, *Gorai.012G119600*) had four isoforms. The gene had four introns and *GrWRKY33_t1* was the dominant isoform. IR event was detected in *GrWRKY33_t2*, *GrWRKY33_t3* and *GrWRKY33_t4* on the second, third and fourth intron, respectively. Among these three IR events, only the last one was detected differential splicing and would be used to verify, however, the other two would not. IR in *GrWRKY33_t3* and *GrWRKY33_t4* introduced PTC caused the second function motif lost. Different from the former, IR in *GrWRKY33_t2* introduced PTC at the 5′ CDS region but not affected the dominant function. RNA-seq data and qRT-PCR revealed that IR event on the fourth introns was decreased under salt stress (Fig. 8c), indicating that salt stress elevated the proportion of function isoforms of *GrWRKY33*.

## Discussion

Plants require controlled systems to defend against various stresses. Previous studies have reported many genes that are responsive to stresses at the transcriptional level [45, 46], however, gene regulation at the posttranscriptional level was less investigated under stress, especially for salt stress. Since Berget et al. [47] discovered intervening sequences, an increasing amount of evidence has revealed that AS plays an important role in transcription regulation and contributes to the functional diversity of eukaryotic genomes. AS is involved in many physiological processes, including the responses to biotic and abiotic stresses [12, 15, 16]. Most of the AS events that have been found to be involved in the response to abiotic stress are linked to genes with regulatory roles, covering all levels of the regulation of gene expression [48]. Nevertheless, large-scale or genome-wide studies of AS dynamics under salt stress conditions are still relatively scarce. In this study, through comprehensive transcriptome analysis of high-throughput RNA-seq data, we revealed genome-wide AS events under salt stress in *G. davidsonii*. Our analysis suggests that 31.58% of the multi-exonic genes in the *G. davidsonii* genome are alternatively spliced under salt-stress conditions. Furthermore, four basic AS events was increased post salt stress, and we have identified DAS genes associated with several biological processes, such as “response to salt stress”, “water transport” and “metabolic process”. Moreover, we observed that genes encoding splicing factors are frequently alternatively spliced under salt stress.

### An overview of AS in *G. davidsonii* under salt stress

In *G. raimondii*, 16,437 AS events involving 77,569 unique transcripts at 10,197 genes have been identified, indicating that ~ 32% of the multi-exonic genes had at least one AS event [7]. In this study, 14,172 AS events involving 6798 genes were identified in *G. davidsonii* suggested that 31.58% of the multi-exonic genes were alternatively spliced. The ratio of alternative splicing genes is lower than other plants [22–24, 30] suggests difference between cotton and other plants. We also found that IR was less frequent and AA was more frequent in *G. davidsonii* compared with that in *G. raimondii*, implying interspecific differences in the types of AS events in the two diploid D-genome cotton species. Actually, the differences in alternative splicing have also been described between different ecotypes of *Arabidopsis* [49] and *Vitis vinifera* [31], giving support to the argument that changes in splicing may contribute to the evolutionary adaptation process. Furthermore, most AS events were tissue or stress specific, only 17% of total events were commonly detected in all four tissues/treatments conditions in *G. davidsonii* (Fig. 3a). Similar AS profiles are also observed in other plants, such as *Arabidopsis* [23], *Vitis vinifera* [31] and *Zea mays* [50]. The result implied that AS may contribute to the function diversity in the tissue or stress-specific genes.

### AS was regulated in response to salt stress

We found that AS events were increased under salt stress no matter in roots or in leaves. This finding is consistent with the previous studies on AS under environmental stresses [23, 48]. These increased AS events may lead to a wider plasticity for plants to enable them to adapt to various stresses. To further understand the gene function affected by alternative splicing under salt stress, we performed gene ontology (GO) analysis of DAS and found that they were enriched in some biological processes, such as “response to salt stress”, “water transport” and “metabolic process”. This result further suggested that the DAS induced by salt stress could regulate the salt response. An interesting finding is that “response to calcium ion” is not only overrepresented in our study, but also frequently overrepresented in other plants [23] or other stress condition [32]. Calcium-signaling pathway plays a crucial role in stress response. Exposure to salinity activates the Salt Overly Sensitive (SOS) pathway, leading to Ca^2+^-dependent increased activity of SOS1, a plasma membrane Na^+^-H^+^ antiporter that enables adaptation through Na^+^ efflux [51, 52]. We suspect that Calcium regulation pathway is sensitive to stress condition at splicing level. Some important salt stress response genes or transcript factors, such as KCO, PIP and WRKY, were regulated by AS. The expression of these genes may have three fates, that decreased the abundance of function isoforms by upregulated the alternative splicing isoforms like *GrPIP2.7*, that increased the abundance of function isoforms by downregulated the alternative splicing isoforms like *GrWRKY33*, and that remained the abundance with AS events occur at 5′ UTR region that may not affect the translation of CDS region like *GrKCO1*. In addition, DAS genes induced by salt stress were enriched in pathways related to RNA splicing, indicating that AS events of the RNA splicing-related genes are induced by salt stress and regulated by themselves or other splicing factors [53, 54]. These AS events may introduce new domain and subsequently impact the function of gene [55, 56]. Consequently, AS is a complex and important regulatory mechanism in response to salt stress in cotton.

### SR splicing factors were regulated by AS under salt stress

The splicing of introns from pre-mRNA is carried out by one of the largest molecular complexes of the cell, the spliceosome, which consists of five small nuclear ribonucleoproteins (snRNPs) and numerous additional proteins [10]. Members of the Serine/arginine (SR) protein family are well-known non-snRNP spliceosomal factors [57]. A few previous studies showed that over-expression of SRs or other splicing factors could increase plant tolerance to salt and other stresses [43, 58–60]. Interestingly, the AS pattern of most SRs has been shown to change under stress conditions [12, 23]. It suggests that pre-mRNAs of SRs are themselves alternatively spliced and this splicing is under tight spatial, temporal, and environmental control. In this study, 29 orthologous SR genes were identified in *G. davidsonii*. Of them, 16 were differential splicing and only two were differential expression in roots or leaves. This result is consistent with the previous report on *Arabidopsis* [23], which show that splicing factors involved in salt stress function mainly via splicing pathways rather than regulated directly by salt stress. Thatcher et al. [30] also demonstrated that the expression level of known splicing factors was not the major driving force behind genotypic AS variation. Therefore, we speculated that pre-mRNAs of SRs were themselves alternatively spliced under salt stress and the diversity of SR splicing products consequently increased the number of novel AS isoforms identified (Figs. 3a and 6b), and SR genes belong to RSZ subfamily might play a crucial role in splicing regulation under salt stress condition in cotton.

### AS plays a crucial role in salt stress response at the posttranscriptional level

The molecular mechanisms of the response to salt stress involve signal transduction pathways [61], transcription factors [62] and genes [63], which have been well documented at the transcriptional level. However, little is known about the regulation of salt stress-specific gene expression at the post-transcriptional level. Here, we found that AS events were abundant in the response to salt stress in *G. davidsonii*. However, one crucial question is whether the increase in AS events is an acclimation response or merely a consequence of splicing errors caused by stress damage. Several previous studies suggested that the stress-induced increase in AS could be ascribed to splicing errors and could weaken the function of the corresponding genes by decreasing the abundance of functional transcripts [23, 64]. However, some evidence showed that AS could promote stress tolerance by increasing proteomic diversity [65, 66]. Most aberrant splicing events (splicing errors) could be removed by mRNA surveillance mechanisms such as nonsense mediated mRNA decay (NMD) [67], thus, some splicing variants, neutral or beneficial to the organism, can be selectively fixed as functional AS events. A large-scale study using 39 million expressed sequence tags from 47 eukaryotic species revealed that the proportion of AS genes and the average number of AS isoforms per gene (AS level) have gradually increased over the past 1.4 billion years, indicating that AS complexity can be considered a strong predictor of organismal complexity [68]. In present study, we found the number of AS events and isoforms increased under salt stress condition (Figs. 3 and 6). In addition, DAS genes were overrepresented in terms of “response to salt stress” and “metabolic process”. We also found that the splicing pattern was diverse in tissues and developmental stage under salt stress (Fig. 7). Compared to the differential expression regulation in our previous study [26], salt stress response at splicing level is more complicated. The specific regulation pathway of response to salt stress is hard to verify only by transcriptome sequence, and further research at molecular level, based on the key genes regulated at splicing level such as *P5CS* and *PIP*, need to be conducted.

## Conclusion

In this study, through comprehensive transcriptome analysis of high-throughput RNA-seq data, we revealed genome-wide AS events under salt stress in *G. davidsonii*. We found that that the number of AS events under stress conditions was significantly higher than that under normal conditions. The functions of these genes related to AS were enriched in several biological processes, such as “response to salt stress”, “water transport” and “metabolic process”. We also detected splicing factors (SFs) involved in AS regulation and found their alternative splicing under salt stress. In addition, several salt response genes, such as *P5CS*, *KCO1*, *PIP* and *WRKY33*, were identified with differential splicing. This study indicates that AS plays the important roles in plant response to salt stress.

## Methods

### Plant material and salt stress conditions

Diploid wild cotton species *G. davidsonii* was used for the study. Following our pervious study, the salt stress concentration of 200 mM NaCl was identified by comparing the salt tolerance of three cotton accessions, diploid wild cotton species *G. davidsonii* and two *G. hirsutum* cultivars, ZS9612 and Z9807, with sensitivities and insensitivities to salinity stress, respectively [26]. All necessary permits for collecting the *G. davidsonii* seeds were obtained from Nanjing Agricultural University, China.

The *G. davidsonii* seeds were surface-sterilized with 70% ethanol for 30–60s and 10% H_2_O_2_ for 60–120 min, followed by washing with sterile water. Sterilized seeds were germinated at 26 °C under long day conditions in a 16 h light/8 h dark cycle with a light intensity of 150 μmol m^− 2^ s^− 1^ on 1/2 MS solid medium. Three days after germination, the plants were transferred to 1/2 Hoagland nutrient solutions at pH 6.0.

The seedlings with two true leaves and one heart-shaped leaf were randomly selected and cultured in 1/2 Hoagland solutions supplemented with 200 mM NaCl for salinity stress treatment. Due to different salt damage mechanisms when plant exposure to salinity [69, 70], five time points, 12, 24, 48, 96, and 144 h after exposure, were set for the leaf and root sampling. All samples were frozen quickly in liquid N_2_ and stored at −70゜C for further use. Plant seedlings grown in normal 1/2 Hoagland nutrient solution was used as controls. All the cotton plants cultured in 1/2 Hoagland solutions were grown in chambers under long day conditions with a 16 h light/8 h dark cycle at 28/25゜C in Nanjing Agricultural University. The 1/2 Hoagland nutrient solution was replaced every day. These evaluations were not relevant to human subject or animal research. Therefore, they did not involve any endangered or protected species.

### RNA extraction, cDNA library preparation, and RNA-seq

Total RNA was extracted from roots and leaves by the cetyltrimethylammonium bromide (CTAB)-sour phenol extraction method [71]. The RNA was digested with RNase-Free DNase (Qiagen) and checked for integrity by capillary gel electrophoresis. Library preparation for RNA-seq was performed using the TruSeq RNA Sample Preparation Kit (Illumina, Cat. NRS–122–2002) with 500 ng of total RNA. Accurate quantitation of cDNA libraries was performed using the QuantiFluor dsDNA System (Promega). The size range of the final cDNA libraries was determined by applying the DNA 1000 chip on the Bioanalyzer 2100 (Agilent; 280 bp). The cDNA libraries were amplified and sequenced using the cBot and HiSeq2000 systems from Illumina. Two biological replicates from each sample were used for all RNA-seq experiments.

### Reads mapping and transcript assembly

After preprocessing the RNA-seq data with an NGS QC toolkit [72], the reads were mapped to the *G. raimondii* genome using a Tophat spliced aligner [73]. Based on default Tophat parameters, four mismatches were allowed for reads mapping. The sequence alignment/map files generated by Tophat were used as the input to the software Cufflinks [74], which assembles the alignments in the sequence alignment/map file into transfrags. Cufflinks does this assembly independently of the existing gene annotations and constructs a minimum set of transcripts that best describes the RNA-seq reads. The unit of measurement used by Cufflinks to estimate transcript abundance is FPKM. The Cufflinks statistical model probabilistically assigns reads to the assembled isoforms. Cuffcompare was used to merge the assemblies with the reference annotation (G.raimondii_221_v2.1.gene.gff) into a single GTF file that was used later to identify alternative splicing (AS) events. The class codes in the Cuffcompare output were used to identify novel isoforms, intergenic transcripts, and splice junctions.

### Novel isoforms prediction and alternative splicing analysis

Using an empirical method [30], 2.6 FPKM was chosen as the expression cutoff for alternative splicing isoforms. In addition, the AS isoforms that were identified in both replications, were regarded as the stable isoforms. We used the ASTALAVISTA v2.2 software [75] with the parameters (-t asta -i) to quantify the type of AS events based on the assembled transcripts by the Cufflinks software. Four basic AS events (IR, AA, AD and ES) were performed and the remaining complex AS events were collectively grouped as other type.

### Differential alternative splicing events analysis

Each AS event consist of constitutive and alternative splicing event (e.g. IR event consist of intron splicing and intron retain). First, FPKM of constitutive and alternative splicing event were calculated by sum of the corresponding isoforms (constitutive or alternative splicing event may have multiple isoforms). Then, fisher’s exact test was applied to above FPKM to analyze differential alternative splicing between well-watered and salt-stress treatments with *p* < 0.05 as significance. In order to assess the change of AS events caused by salt stress, each FPKM of event was then converted into a percentage of total (FPKM (AS events isoforms)/FPKM (total isoforms)) and subsequently calculated the difference.

### Go enrichment

AgriGO software [76] was used for gene ontology analysis, and Singular Enrichment Analysis (SEA) was performed with the statistical method of Fisher’s exact tests. The input sample list was the *G. raimondii* gene ID (http://www.phytozome.net/), which was converted from the original ID of the Cuffdiff default configuration, and the background was whole annotated genes in *G. raimondii*. The output of enrichment needed Benjamini and Hochberg-adjusted *P*-values (FDR) < 0.05.

### RT-PCR and qRT-PCR validation

RT-PCR and qRT-PCR were done using a new set of RNAs extracted with the same tissues and treatment time points as that for RNA-seq analysis. The selected AS events were validated by RT-PCR using a set of primers (Table S6) that were designed based on each AS event. *EF1-α* was used as an internal standard. The amplification reactions were performed under the following conditions: 95 °C for 5 min, followed by 32 cycles of 95 °C for 30 s, 58 °C for 30 s, and 72 °C for 60 s. qRT-PCR validations was performed on three alternative splicing events in the salt stress response genes, using a Bio-Rad CFX96 Real-Time instrument and the light cycler fast start DNA Master SYBR Green I kit (Roche, Basel, Switzerland). Relative abundance of each splicing event by qRT-PCR using primers specific for each splicing variant or common primers for whole transcripts (Table S7). The *GhHis3*, which is a constitutive expression gene in cotton [77], its orthologous gene *GrHis3* in *G. raimondii* was used to design the internal control primers (Table S7) and ΔΔCt method was used for data analysis [78].

## Additional files

Additional file 1: Figure S1. Frequency of the top five most abundant isoforms across the samples. Each line in chart represent a sample with two biological replicates: the roots of well-watered control plants (RC), the roots of salt-stressed plants (RS), the leaves of well-watered controls plants (LC), the leaves of salt-stressed plants (LS) and the subsequent number represent the time point post treatment. (TIFF 452 kb)

Additional file 2: Figure S2. FPKM cutoff used for novel transcripts selection. The loss of expression of known transcripts (false negatives) plotted against the retention of randomly generated artificial transcripts (false positive) at various FPKM (1–10) abundance cutoffs. Fractions represent the number of isoforms found above a given cutoff in at least one library. (TIFF 130 kb)

Additional file 3: Figure S3. The numbers of exon and isoform distribution for high confidence unigenes. A. Number of exons per isoform distribution. B. Number of isoforms per gene distribution. (TIFF 120 kb)

Additional file 4: Table S1. Number of AS events in four groups. (DOCX 14 kb)

Additional file 5: Figure S4. The length distribution of differential alternative splicing events. To AA and AD events, the length more than 50 bp is few and not be shown in the chart. (TIFF 341 kb)

Additional file 6: Figure S5. The length distribution of differential alternative splicing events. To AA and AD events, the length more than 50 bp is few and not be shown in the chart. (TIFF 99 kb)

Additional file 7: Figure S6. The comparison of differential alternative splicing and differential expression genes. RDAS for differential alternative splicing in roots; RDEG for differential expression genes in roots; LDAS for differential alternative splicing in leaves; LDEG for differential expression genes in leaves. (TIFF 299 kb)

Additional file 8: Table S2. Biological process of DAS genes and their corresponding isoforms distribution. (DOCX 15 kb)

Additional file 9: Table S3. DAS genes related to salt stress response. (DOCX 17 kb)

Additional file 10: Table S4. Correlation analysis between frequency change of AS events and fold change of gene expression for salt response genes. (XLSX 32 kb)

Additional file 11: Table S5. Information on SR proteins identified in *G. davidsonii*. (DOCX 14 kb)

Additional file 12: Figure S7. RT-PCR validation of alternative splicing events. Each gene was amplified at the roots of well-watered control, the roots of salt-stressed, the leaves of well-watered controls and the leaves of salt-stressed condition. *EF1-α* was used as an internal standard. (TIFF 279 kb)

Additional file 13: Table S6. Primers used for RT-PCR of alternative splicing events. (DOCX 14 kb)

Additional file 14: Table S7. Primers used for qRT-PCR of differential alternative splicing events. (DOCX 15 kb)

Additional file 15: Figure S8. Validation on DAS events by qRT-PCR. The gene IDs marked in red indicates that the predicted DAS events are unable to be validated. *His3* is a constitutive expression gene in cotton, and *GrHis3* (*Gorai.003G041300*) in *G. raimondii* was used to design primers for the internal control analysis. (TIFF 501 kb)

## Abbreviations

AA: Alternative acceptor site
AD: Alternative donor site
AS: Alternative splicing
DAS: Differential alternative splicing
DEG: Differential expression genes
ES: Exon skipping
FPKM: Fragments per kilobase per million reads
GO: Gene Ontology
hnRNP: heterogeneous nuclear ribonucleoprotein particle
IR: Intron retention
LC: The leaves of well-watered controls plants
LS: The leaves of salt-stressed plants
NMD: Nonsense mediated mRNA decay
PTC: Premature termination codons
RBPs: RNA binding proteins
RC: The roots of well-watered control plants
ROS: Reactive oxygen species
RS: The roots of salt-stressed plants
SFs: Splicing factors
snRNPs: small nuclear ribonucleoprotein particles
SOS: Salt overly sensitive
SR: Ser/Arg-rich
UV: Ultraviolet
