# Supplementary figures and images for: RNA-seq analysis reveals alternative splicing under salt stress in cotton, Gossypium davidsonii
Source: BMC Genomics. 2018 Jan 23;19:73. doi: 10.1186/s12864-018-4449-8 (PMC5782385; doi:10.1186/s12864-018-4449-8)

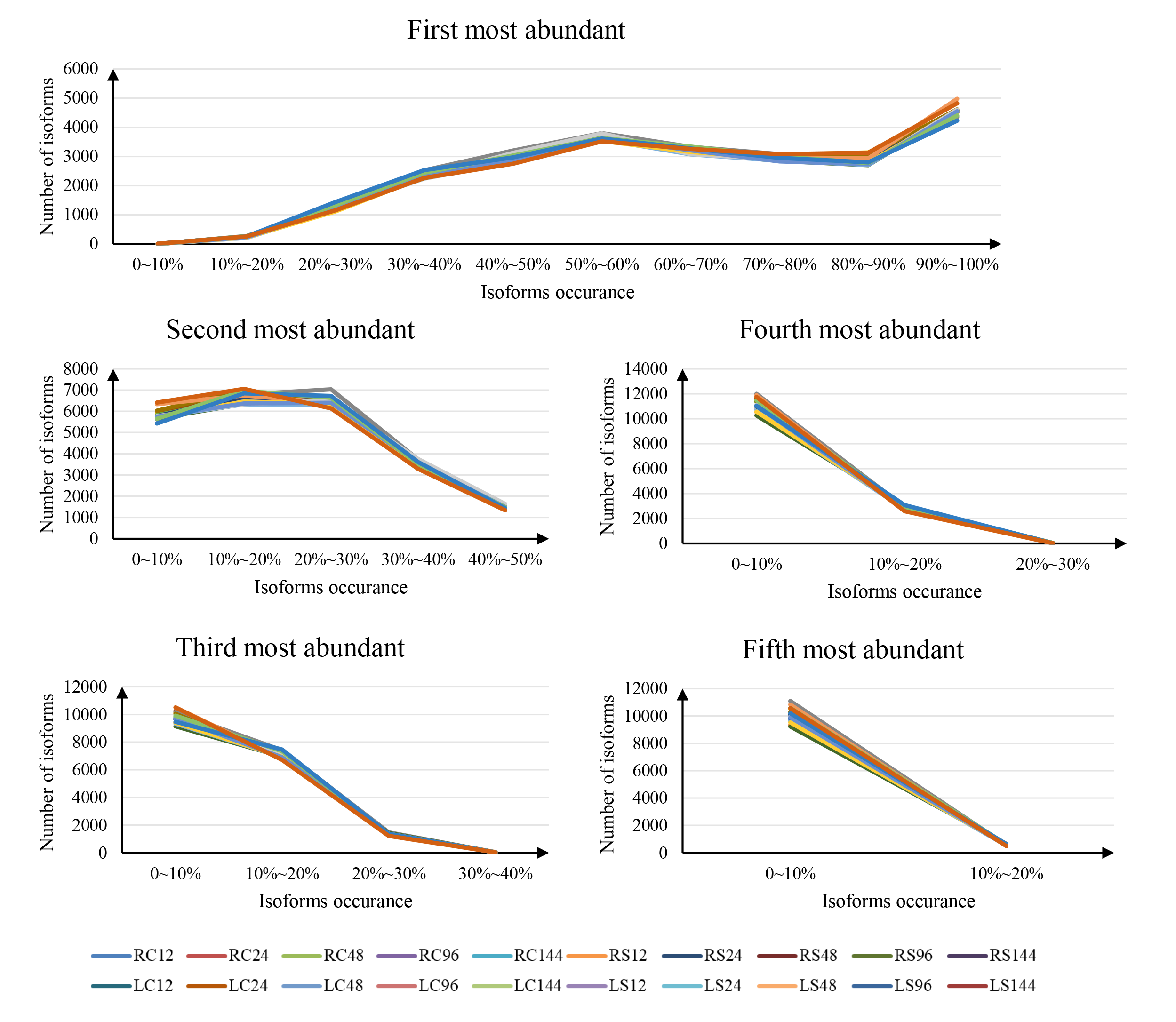

Supplement: Supplementary file 1 — Frequency of the top five most abundant isoforms across the samples. Each line in chart represent a sample with two biological replicates: the roots of well-watered control plants (RC), the roots of salt-stressed plants (RS), the leaves of well-watered controls plants (LC), the leaves of salt-stressed plants (LS) and the subsequent number represent the time point post treatment. (TIFF 452 kb) [file 12864_2018_4449_MOESM1_ESM.tif]

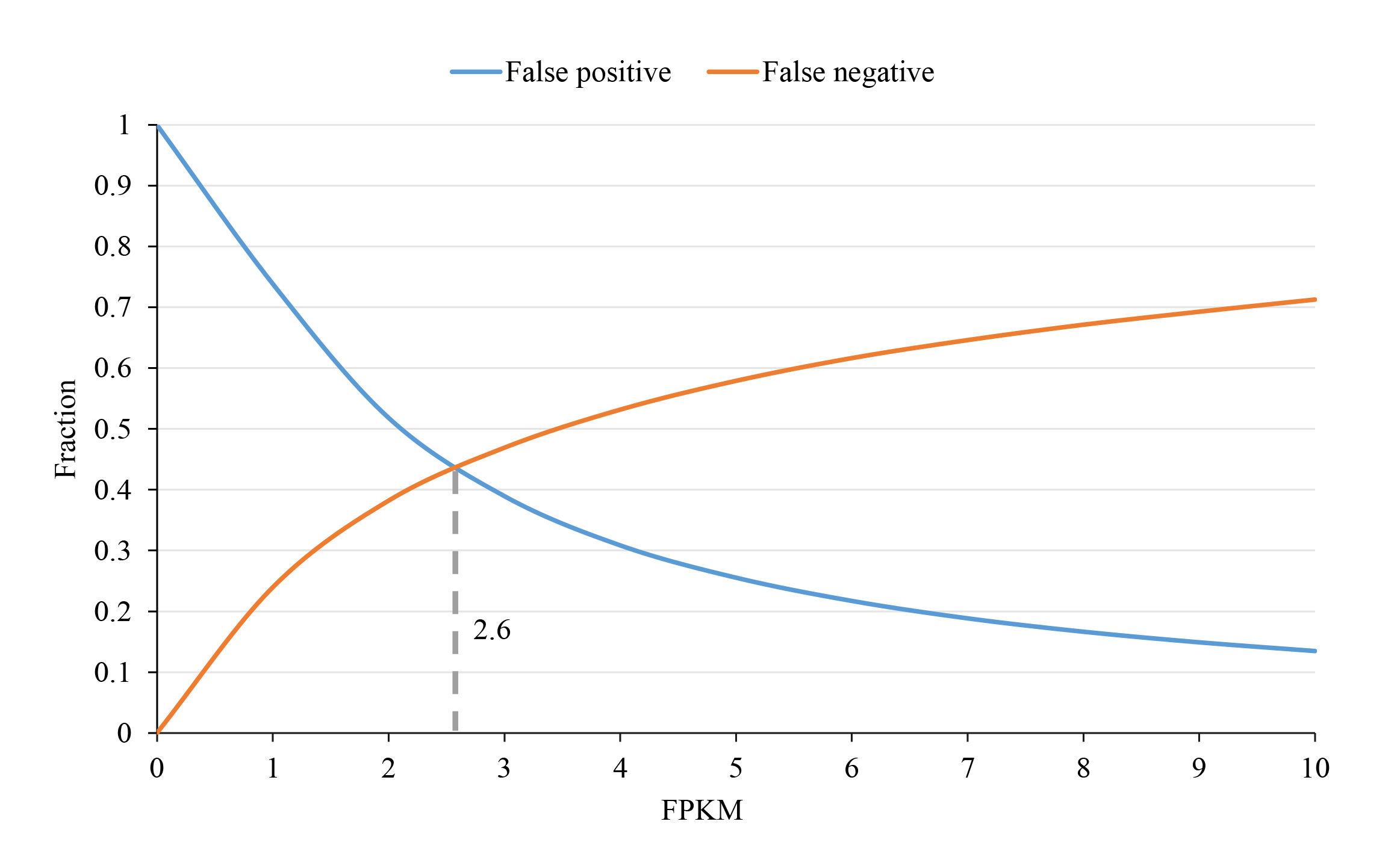

Supplement: Supplementary file 2 — FPKM cutoff used for novel transcripts selection. The loss of expression of known transcripts (false negatives) plotted against the retention of randomly generated artificial transcripts (false positive) at various FPKM (1–10) abundance cutoffs. Fractions represent the number of isoforms found above a given cutoff in at least one library. (TIFF 130 kb) [file 12864_2018_4449_MOESM2_ESM.tif]

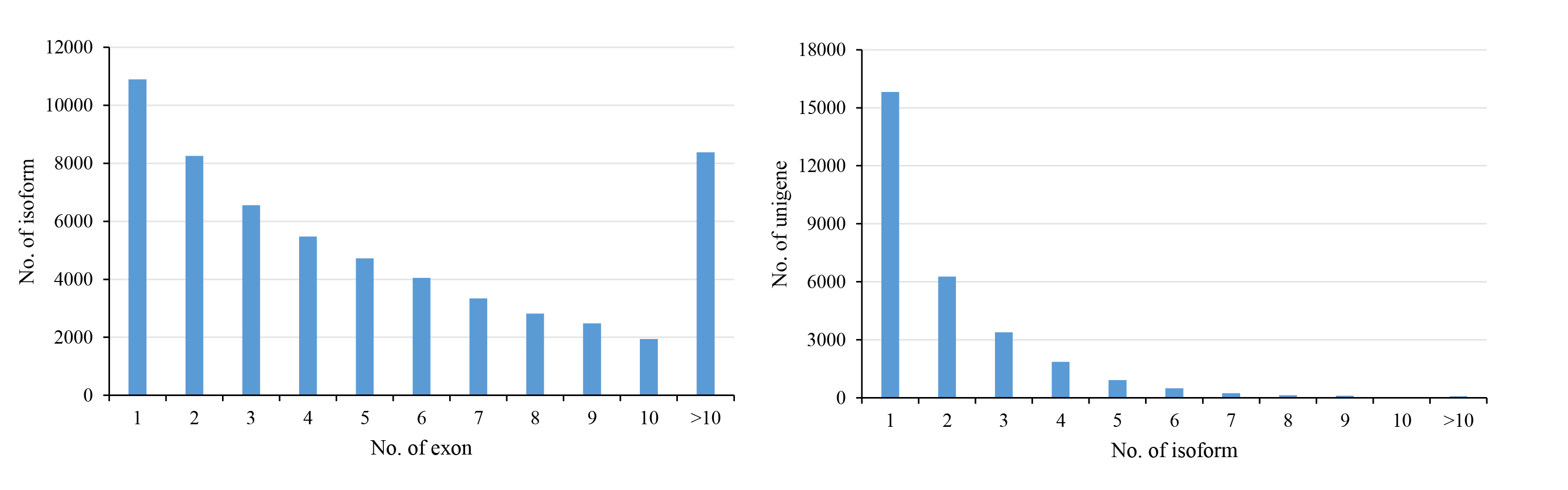

Supplement: Supplementary file 3 — The numbers of exon and isoform distribution for high confidence unigenes. A. Number of exons per isoform distribution. B. Number of isoforms per gene distribution. (TIFF 120 kb) [file 12864_2018_4449_MOESM3_ESM.tif]

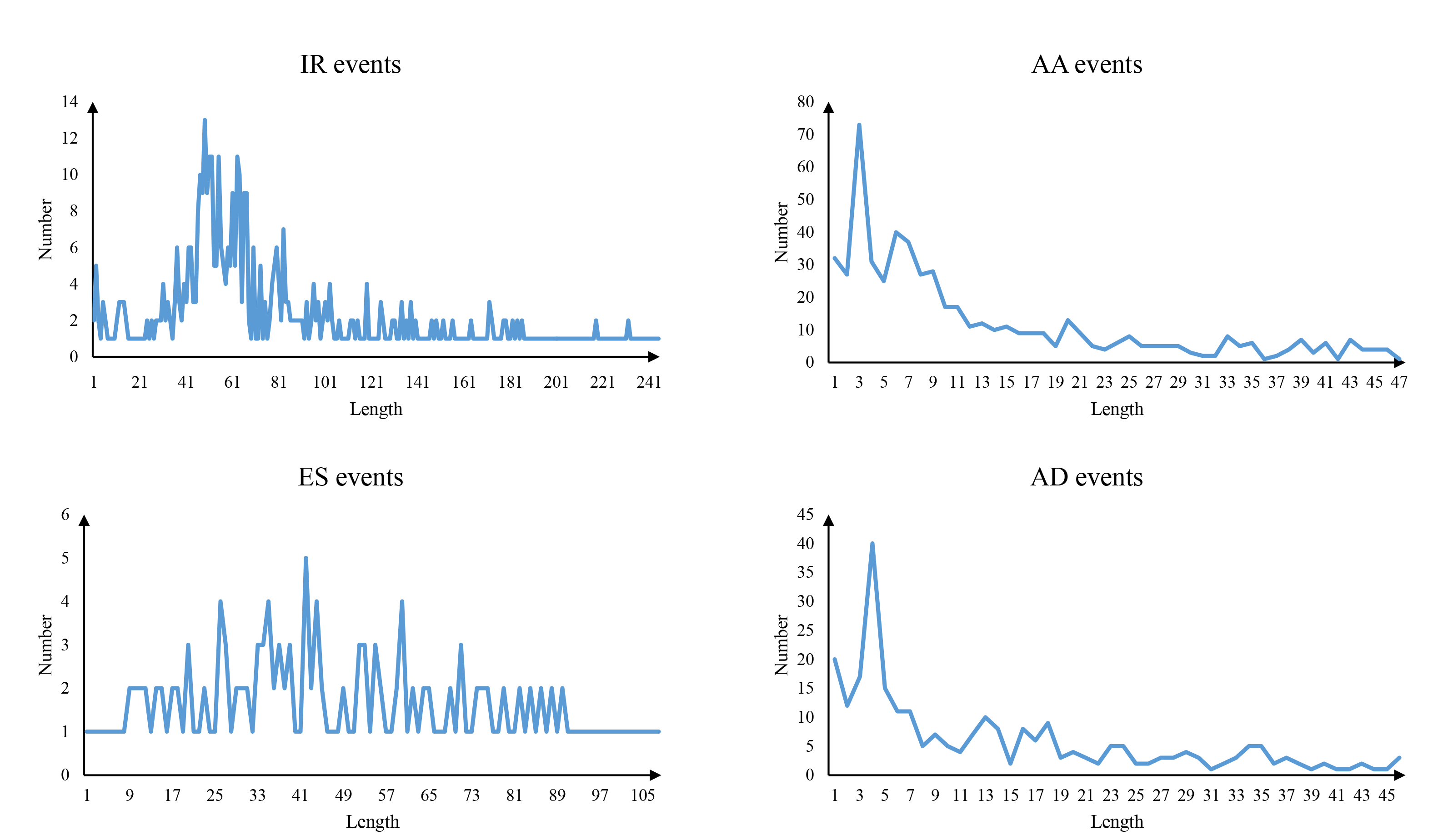

Supplement: Supplementary file 5 — The length distribution of differential alternative splicing events. To AA and AD events, the length more than 50 bp is few and not be shown in the chart. (TIFF 341 kb) [file 12864_2018_4449_MOESM5_ESM.tif]

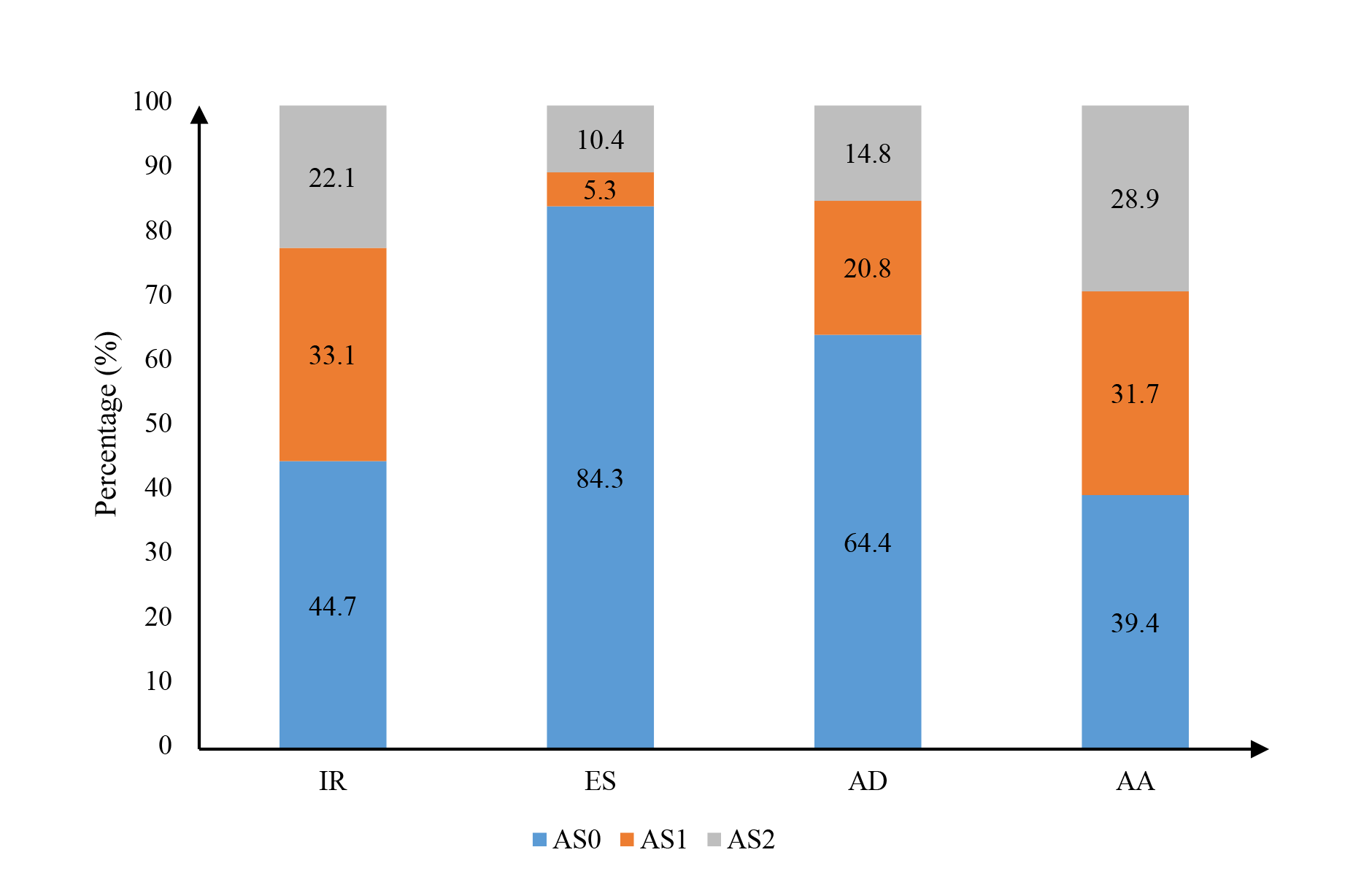

Supplement: Supplementary file 6 — The length distribution of differential alternative splicing events. To AA and AD events, the length more than 50 bp is few and not be shown in the chart. (TIFF 99 kb) [file 12864_2018_4449_MOESM6_ESM.tif]

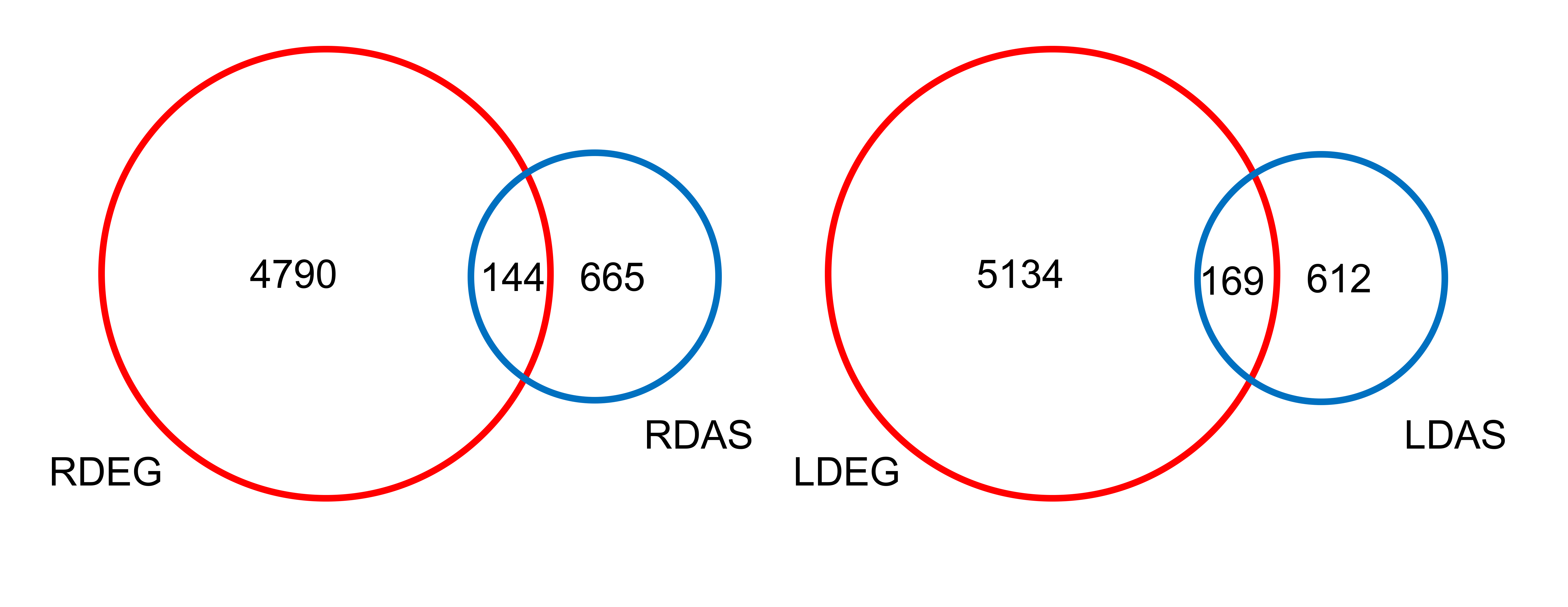

Supplement: Supplementary file 7 — The comparison of differential alternative splicing and differential expression genes. RDAS for differential alternative splicing in roots; RDEG for differential expression genes in roots; LDAS for differential alternative splicing in leaves; LDEG for differential expression genes in leaves. (TIFF 299 kb) [file 12864_2018_4449_MOESM7_ESM.tif]

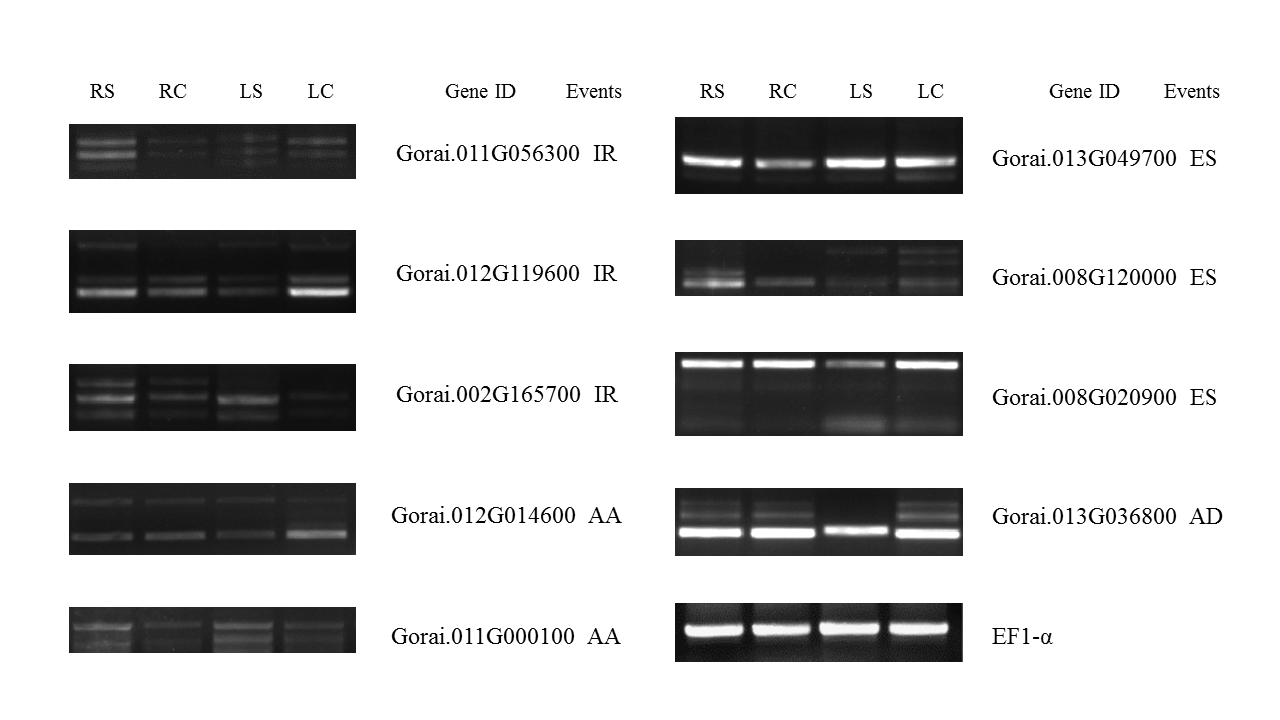

Supplement: Supplementary file 12 — RT-PCR validation of alternative splicing events. Each gene was amplified at the roots of well-watered control, the roots of salt-stressed, the leaves of well-watered controls and the leaves of salt-stressed condition. EF1-α was used as an internal standard. (TIFF 279 kb) [file 12864_2018_4449_MOESM12_ESM.tif]

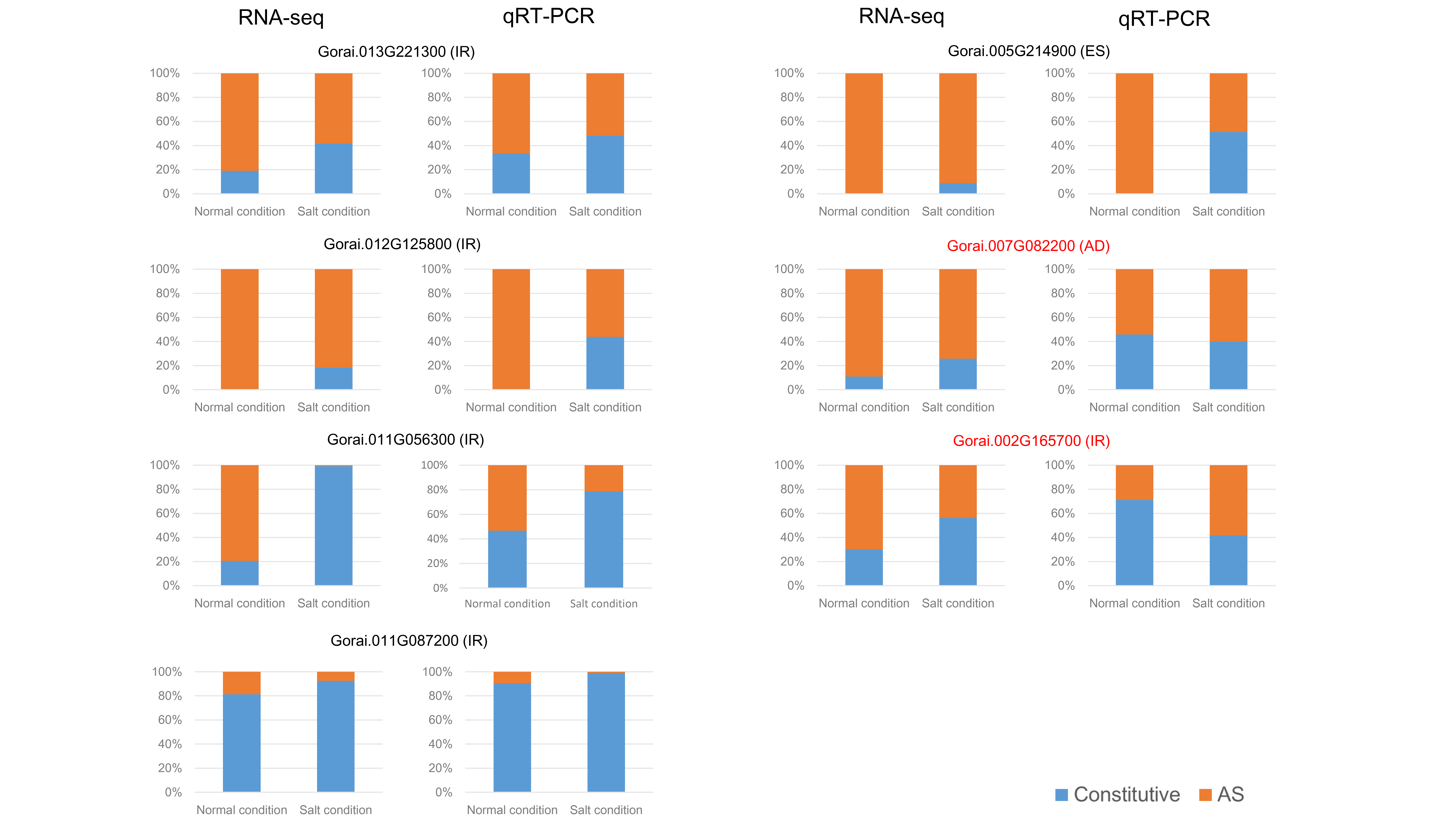

Supplement: Supplementary file 15 — Validation on DAS events by qRT-PCR. The gene IDs marked in red indicates that the predicted DAS events are unable to be validated. His3 is a constitutive expression gene in cotton, and GrHis3 (Gorai.003G041300) in G. raimondii was used to design primers for the internal control analysis. (TIFF 501 kb) [file 12864_2018_4449_MOESM15_ESM.tif]
