# Supplementary material for: RNA-seq analysis reveals alternative splicing under salt stress in cotton, Gossypium davidsonii
Source: BMC Genomics. 2018 Jan 23;19:73. doi: 10.1186/s12864-018-4449-8 (PMC5782385; doi:10.1186/s12864-018-4449-8)
Supplement: Supplementary file 4 — Number of AS events in four groups. (DOCX 14 kb) [file 12864_2018_4449_MOESM4_ESM.docx]

| **Group** | **IR** | **AA** | **AD** | **ES** | **Other** |
| --- | --- | --- | --- | --- | --- |
| RC | 2755 | 2448 | 972 | 614 | 535 |
| RS | 3030 | 2636 | 1037 | 694 | 570 |
| LC | 3061 | 2522 | 1052 | 607 | 625 |
| LS | 3193 | 2654 | 1129 | 617 | 676 |
| Total | 5063 | 4146 | 1809 | 1093 | 2061 |

**Table S1 Number of AS events in four groups**

RC: root well-watered control; RS: root salt-stressed treatment; LC: leaf well-watered control; LS: leaf salt-stressed treatment

AA: alternative acceptor site; AD: alternative donor site; ES: exon skipping; IR: intron retention.
