# Supplementary material for: RNA-seq analysis reveals alternative splicing under salt stress in cotton, Gossypium davidsonii
Source: BMC Genomics. 2018 Jan 23;19:73. doi: 10.1186/s12864-018-4449-8 (PMC5782385; doi:10.1186/s12864-018-4449-8)
Supplement: Supplementary file 8 — Biological process of DAS genes and their corresponding isoforms distribution. (DOCX 15 kb) [file 12864_2018_4449_MOESM8_ESM.docx]

| **Term** | **Query item(roots)** | **Log10(1/FDR) (roots)** | **Query item(leaves)** | **Log10(1/FDR) (leaves)** | **No. of isoforms in RC** | **No. of isoforms in RS** | **No. of isoforms in LC** | **No. of isoforms in LS** |
| --- | --- | --- | --- | --- | --- | --- | --- | --- |
| response to cadmium ion | 42 | 12.06 | 34 | 7.55 | 191 | 195 | 150 | 164 |
| response to salt stress | 40 | 10.88 | 40 | 8.19 | 180 | 190 | 155 | 170 |
| response to osmotic stress | 41 | 10.10 | 43 | 12.00 | 180 | 190 | 155 | 170 |
| response to stimulus | 152 | 9.34 | 149 | 17.46 | 605 | 617 | 500 | 524 |
| response to oxidative stress | 22 | 5.31 | 17 | 17.59 | 116 | 122 | 97 | 105 |
| water transport | 14 | 6.17 | 13 | 6.00 | 86 | 91 | 70 | 80 |
| ion homeostasis | 10 | 5.92 | 12 | 6.52 | 117 | 121 | 171 | 172 |
| catabolic process | 91 | 4.85 | 97 | 3.54 | 386 | 412 | 436 | 452 |
| metabolic process | 246 | 4.02 | 270 | 2.13 | 931 | 960 | 803 | 841 |
| protein catabolic process | 27 | 3.30 | 22 | 2.92 | 110 | 118 | 96 | 106 |
| glycolysis | 29 | 3.00 | 25 | 6.38 | 180 | 187 | 233 | 238 |
| glucose metabolic process | 42 | 2.00 | 56 | 3.89 | 194 | 208 | 263 | 264 |
| alcohol metabolic process | 51 | 1.22 | 79 | 0.08 | 267 | 275 | 365 | 375 |
| RNA splicing, via transesterification reactions | 8 | 2.52 | 6 | 1.30 | 52 | 55 | 53 | 56 |
| RNA splicing | 13 | 1.63 | 9 | 0.38 | 68 | 76 | 75 | 74 |
| translation | 24 | 0.48 | 29 | 1.41 | 102 | 110 | 90 | 90 |
| photosynthesis, light reaction | 0 | 0.00 | 25 | 4.72 | 7 | 7 | 25 | 24 |
| photosynthesis | 0 | 0.00 | 27 | 3.80 | 7 | 7 | 25 | 24 |
| chlorophyll metabolic process | 5 | 0.00 | 17 | 3.54 | 156 | 176 | 250 | 244 |

**Table S2 Biological process of DAS genes and their corresponding isoforms distribution**
