# Supplementary material for: RNA-seq analysis reveals alternative splicing under salt stress in cotton, Gossypium davidsonii
Source: BMC Genomics. 2018 Jan 23;19:73. doi: 10.1186/s12864-018-4449-8 (PMC5782385; doi:10.1186/s12864-018-4449-8)
Supplement: Supplementary file 9 — DAS genes related to salt stress response. (DOCX 17 kb) [file 12864_2018_4449_MOESM9_ESM.docx]

**Table S3 DAS genes related to salt stress response**

| **Cotton gene id** | **Arabidopsis id** | **Gene symbol** | **Description** | **AS category** |
| --- | --- | --- | --- | --- |
| Gorai.013G153400 | AT5G55630.1 | KCO1,TPK1 | Outward rectifying potassium channel protein | AD |
| Gorai.009G186800 | AT1G20693.2 | HMGB2 | high mobility group B2 | IR |
| Gorai.009G288900 | AT1G22640.1 | MYB3 | myb domain protein 3 | AA |
| Gorai.013G211700 | AT4G31300.1 | PBA1 | N-terminal nucleophile aminohydrolases (Ntn hydrolases) superfamily protein | ES, AA |
| Gorai.003G095000 | AT5G62530.1 | ALDH12A1 | aldehyde dehydrogenase 12A1 | ES |
| Gorai.008G141100 | AT2G47470.1 | PDI11,UNE5 | thioredoxin family protein | AA |
| Gorai.011G005000 | AT1G30580.1 | - | GTP binding | ES |
| Gorai.011G154000 | AT4G13940.1 | SAHH1 | S-adenosyl-L-homocysteine hydrolase | IR |
| Gorai.011G083000 | AT1G52570.1 | PLDALPHA2 | phospholipase D alpha 2 | IR |
| Gorai.002G254700 | - | - | - | other |
| Gorai.012G119600 | AT2G38470.1 | WRKY33 | WRKY DNA-binding protein 33 | IR |
| Gorai.011G087200 | - | - | - | IR |
| Gorai.008G029100 | AT4G39260.3 | GRP8 | cold, circadian rhythm, and RNA binding 1 | IR |
| Gorai.013G046000 | AT1G49760.1 | PAB8 | poly(A) binding protein 8 | AD |
| Gorai.004G235100 | AT2G30520.1 | RPT2 | Phototropic-responsive NPH3 family protein | IR |
| Gorai.004G256400 | AT3G42050.1 | - | vacuolar ATP synthase subunit H family protein | ES, other |
| Gorai.012G082900 | AT1G12840.1 | DET3 | vacuolar ATP synthase subunit C (VATC) / V-ATPase C subunit / vacuolar proton pump C subunit (DET3) | ES |
| Gorai.009G283800 | AT1G21750.1 | PDI5,PDIL1-1 | PDI-like 1-1 | AD |
| Gorai.009G407500 | AT2G36460.1 | - | Aldolase superfamily protein | IR, other |
| Gorai.008G228600 | AT4G18010.2 | 5PTASE2 | myo-inositol polyphosphate 5-phosphatase 2 | ESAA |
| Gorai.009G224800 | AT1G50010.1 | TUA2 | tubulin alpha-2 chain | IR |
| Gorai.005G173600 | AT1G24460.1 | - | - | AA |
| Gorai.007G150200 | AT2G20360.1 | - | NAD(P)-binding Rossmann-fold superfamily protein | IR, AD, other |
| Gorai.011G283200 | AT4G00430.1 | PIP1;4 | plasma membrane intrinsic protein 1;4 | AA, other |
| Gorai.011G099300 | AT2G18040.1 | PIN1AT | peptidylprolyl cis/trans isomerase, NIMA-interacting 1 | IR |
| Gorai.012G107700 | AT3G55610.1 | P5CS2 | delta 1-pyrroline-5-carboxylate synthase 2 | AA |
| Gorai.010G088500 | AT3G45310.1 | - | Cysteine proteinases superfamily protein | IR |
| Gorai.013G147100 | AT1G54340.1 | ICDH | isocitrate dehydrogenase | AA |
| Gorai.008G061900 | AT4G35830.1 | ACO1 | aconitase 1 | AA |
| Gorai.011G098100 | AT4G35100.1 | PIP2;7 | plasma membrane intrinsic protein 3 | AA |
| Gorai.009G137300 | AT3G16350.1 | - | Homeodomain-like superfamily protein | IR, AD |
| Gorai.010G003000 | AT4G24190.1 | HSP90.7 | Chaperone protein htpG family protein | other |
| Gorai.013G036800 | AT2G21660.1 | GRP7 | cold, circadian rhythm, and rna binding 2 | IR, AD, other |
| Gorai.013G252200 | AT2G13360.1 | AGT | alanine:glyoxylate aminotransferase | AA, other |
| Gorai.013G192900 | AT1G08830.1 | CSD1 | copper/zinc superoxide dismutase 1 | ES |
| Gorai.001G180000 | AT2G20420.1 | - | ATP citrate lyase (ACL) family protein | other |
| Gorai.007G074000 | AT1G13440.1 | GAPC2 | glyceraldehyde-3-phosphate dehydrogenase C2 | IR, ES, AA |
| Gorai.006G158500 | AT2G36530.1 | ENO2,LOS2 | Enolase | AA, AD |
| Gorai.006G100700 | AT5G40810.1 | - | Cytochrome C1 family | AD, other |
| Gorai.N001600 | AT5G39740.1 | RPL5B | ribosomal protein L5 B | other |
| Gorai.010G091000 | AT5G20720.1 | CPN21 | chaperonin 20 | IR, AA |
| Gorai.011G002600 | AT1G78900.1 | VHA-A | vacuolar ATP synthase subunit A | IR |
| Gorai.013G181000 | AT1G54130.1 | RSH3 | RELA/SPOT homolog 3 | AA |
| Gorai.006G209400 | AT4G23100.1 | GSH1 | glutamate-cysteine ligase | AA |
| Gorai.004G172400 | AT5G05170.1 | CESA3 | Cellulose synthase family protein | IR, AD |
| Gorai.009G054500 | AT5G58070.1 | TIL | temperature-induced lipocalin | AD |
| Gorai.013G159800 | AT3G20390.1 | - | endoribonuclease L-PSP family protein | AA |
| Gorai.010G029800 | AT5G49630.1 | AAP6 | amino acid permease 6 | AA, AD |
| Gorai.004G146800 | AT1G02500.1 | SAM1 | S-adenosylmethionine synthetase 1 | other |
| Gorai.009G071200 | AT3G45140.1 | LOX2 | lipoxygenase 2 | AA, other |
| Gorai.008G280100 | AT5G52060.1 | BAG1 | BCL-2-associated athanogene 1 | AA |
| Gorai.009G396900 | AT2G35940.1 | BLH1 | BEL1-like homeodomain 1 | IR, AA, other |
| Gorai.013G269200 | AT1G08110.4 | - | lactoylglutathione lyase family protein / glyoxalase I family protein | ES |
| Gorai.009G358200 | AT1G66340.1 | EIN1,ETR1 | Signal transduction histidine kinase, hybrid-type, ethylene sensor | ES |
| Gorai.009G237900 | AT1G35720.1 | ANNAT1 | annexin 1 | IR |
| Gorai.013G145100 | AT5G10860.1 | - | Cystathionine beta-synthase (CBS) family protein | AD |
| Gorai.010G174800 | AT1G54100.1 | ALDH7B4 | aldehyde dehydrogenase 7B4 | IR, ES, AA, AD |
| Gorai.011G201100 | AT5G17310.2 | UGP2 | UDP-glucose pyrophosphorylase 2 | IR |
