# Supplementary material for: RNA-seq analysis reveals alternative splicing under salt stress in cotton, Gossypium davidsonii
Source: BMC Genomics. 2018 Jan 23;19:73. doi: 10.1186/s12864-018-4449-8 (PMC5782385; doi:10.1186/s12864-018-4449-8)
Supplement: Supplementary file 11 — Information on SR proteins identified in G. davidsonii. (DOCX 14 kb) [file 12864_2018_4449_MOESM11_ESM.docx]

**Table S5 Information on SR proteins identified in *G. davidsonii***

| **Cotton gene id** | ***Arabidopsis* gene id** | **Gene symbol** | **Description** |
| --- | --- | --- | --- |
| Gorai.002G208700 | AT3G49430 | SRp34a | SER/ARG-rich protein 34A |
| Gorai.006G203400 | AT3G49430 | SRp34a | SER/ARG-rich protein 34A |
| Gorai.007G017000 | AT3G49430 | SRp34a | SER/ARG-rich protein 34A |
| Gorai.010G203100 | AT3G53500 | RSZ32 | RNA-binding (RRM/RBD/RNP motifs) family protein with retrovirus zinc finger-like domain |
| Gorai.011G242700 | AT3G53500 | RSZ32 | RNA-binding (RRM/RBD/RNP motifs) family protein with retrovirus zinc finger-like domain |
| Gorai.013G024100 | AT3G53500 | RSZ32 | RNA-binding (RRM/RBD/RNP motifs) family protein with retrovirus zinc finger-like domain |
| Gorai.010G201600 | AT3G55460 | SCL30 | SC35-like splicing factor 30 |
| Gorai.011G262000 | AT3G55460 | SCL30 | SC35-like splicing factor 30 |
| Gorai.004G044100 | AT3G61860 | RSP31 | RNA-binding (RRM/RBD/RNP motifs) family protein |
| Gorai.008G127200 | AT3G61860 | RSP31 | RNA-binding (RRM/RBD/RNP motifs) family protein |
| Gorai.002G165700 | AT4G31580 | SRZ22 | serine/arginine-rich 22 |
| Gorai.005G183600 | AT4G31580 | SRZ22 | serine/arginine-rich 22 |
| Gorai.009G066600 | AT4G31580 | SRZ22 | serine/arginine-rich 22 |
| Gorai.010G121100 | AT4G31580 | SRZ22 | serine/arginine-rich 22 |
| Gorai.010G245400 | AT4G31580 | SRZ22 | serine/arginine-rich 22 |
| Gorai.005G220000 | AT5G64200 | SC35 | ortholog of human splicing factor SC35 |
| Gorai.009G210200 | AT5G64200 | SC35 | ortholog of human splicing factor SC35 |
| Gorai.012G141900 | AT5G64200 | SC35 | ortholog of human splicing factor SC35 |
| Gorai.013G061300 | AT5G64200 | SC35 | ortholog of human splicing factor SC35 |
| Gorai.007G128300 | AT1G02840 | SRP34 | RNA-binding (RRM/RBD/RNP motifs) family protein |
| Gorai.011G107200 | AT1G09140 | ATSRP30 | SERINE-ARGININE PROTEIN 30 |
| Gorai.013G126300 | AT1G09140 | ATSRP30 | SERINE-ARGININE PROTEIN 30 |
| Gorai.013G205700 | AT1G09140 | ATSRP30 | SERINE-ARGININE PROTEIN 30 |
| Gorai.002G252500 | AT2G37340 | RSZ33 | arginine/serine-rich zinc knuckle-containing protein 33 |
| Gorai.012G091800 | AT2G37340 | RSZ33 | arginine/serine-rich zinc knuckle-containing protein 33 |
| Gorai.009G393200 | AT3G13570 | SCL30A | SC35-like splicing factor 30A |
| Gorai.010G126300 | AT3G13570 | SCL30A | SC35-like splicing factor 30A |
| Gorai.008G280300 | AT4G25500 | RSP35 | arginine/serine-rich splicing factor 35 |
| Gorai.011G183900 | AT5G18810 | SCL28 | SC35-like splicing factor 28 |
