# Supplementary material for: RNA-seq analysis reveals alternative splicing under salt stress in cotton, Gossypium davidsonii
Source: BMC Genomics. 2018 Jan 23;19:73. doi: 10.1186/s12864-018-4449-8 (PMC5782385; doi:10.1186/s12864-018-4449-8)
Supplement: Supplementary file 13 — Primers used for RT-PCR of alternative splicing events. (DOCX 14 kb) [file 12864_2018_4449_MOESM13_ESM.docx]

**Table S6 Primers used for RT-PCR of alternative splicing events.**

| **Gene ID** | **Events** | | **Forward primer (5’-3’)** | **Reverse primer (5’-3’)** |
| --- | --- | --- | --- | --- |
| Gorai.011G056300 | | IR | AACACAGGATGGTGGAAACC | TGATGCACCCACACTCAAAA |
| Gorai.012G119600 | | IR | AGTGGTGCAAACAACAAGTG | CTAACTCCCGAGTTGGGGTA |
| Gorai.002G165700 | | IR | TTCTACTCGTCATCGCTCCT | TGTCAGTAAAGGTTGTGGCG |
| Gorai.012G014600 | | AA | TGGGTGTTCCAAGTGACTAC | CACTCCCCTTGTTCCTCCTA |
| Gorai.011G000100 | | AA | AAAAGCGGAGGTGGAAGTTT | GTCAATTCCACAGGCTGCTA |
| Gorai.013G049700 | | ES | AAGCCTCCTTTTGCCTTTCA | GCAAGCTAGCATTAACCTTTCA |
| Gorai.008G120000 | | ES | TCCCTGTCTTCAACTACTCCA | CAGTCACAGGGATCTCAAGC |
| Gorai.008G020900 | | ES | TCCTTTCTCTCCCTCTGCAA | TAAGCTCCCTCGGAATGGAT |
| Gorai.013G036800 | | AD | TCTGCTCCTCTTAGTTTCCC | GTAACCACCGTTTCCTCCTC |
| Gorai.002G121500 (*EF1-α*) | | control | AGACCACCAAGTACTACTGCAC | CCACCAATCTTGTACACATCC |
