# Supplementary material for: RNA-seq analysis reveals alternative splicing under salt stress in cotton, Gossypium davidsonii
Source: BMC Genomics. 2018 Jan 23;19:73. doi: 10.1186/s12864-018-4449-8 (PMC5782385; doi:10.1186/s12864-018-4449-8)
Supplement: Supplementary file 14 — Primers used for qRT-PCR of differential alternative splicing events. (DOCX 15 kb) [file 12864_2018_4449_MOESM14_ESM.docx]

**Table S7 Primers used for qRT-PCR of differential alternative splicing events.**

| **Gene ID** | **Gene name** | **Primer loci** | **Forward primer (5’-3’)** | **Reverse primer (5’-3’)** |
| --- | --- | --- | --- | --- |
| Gorai.011G098100 | *GrPIP2.7* | AA | TACACCGTTTTCTCAGCCAC | ATCCTATTGGCAAGGGAGC |
|  |  | exon | CGCTTGTTGGACATGGAAGA | CCGATGACAGTGGCAACTAA |
| Gorai.013G153400 | *GrKCO1* | AD | GGTAAAGAATCTTAGCTTAATACCA | TCGAGGAGATTACAACAATTCA |
|  |  | exon | AAGTGGCTCGGACTGTACTA | CCCTCTGATGTGGTTTCTCA |
| Gorai.012G119600 | *GrWRKY33* | IR | CCAAGGTTAGTCATTATTCTCCACC | GCTCCTGTATAAAAGATTTTGACGA |
|  |  | exon | ACAACCACAATGTTCCTGCT | CATGTTTGCAGTTACAGCCG |
| Gorai.013G221300 | *GrCID7* | IR | GCACTTGGTTCCTCGTGATG | TGGAAGTGATGGATATGGAACG |
|  |  | exon | ACCCCTAGCAAGGTAACAACT | CTTGTTGCATCAGCCGCAAT |
| Gorai.012G125800 | *-* | IR | TGATTTGGATGGAGATTACGGT | ACACCAAATATCGAGGTGATGACT |
|  |  | exon | TGACGTAAGCCTGAAGTGGAG | GTGGGTTAGCTGAGGGGTAAG |
| Gorai.011G056300 | *GrRAP2.12* | IR | AGGAGGGTGTTTTCTGAGCGA | GAATAATAAAGCAGCTGCCAGAGG |
|  |  | exon | GCTGCTTTATTATTCCAGTTTGTGT | CTTCAACTTCACAACAGATTTTGAC |
| Gorai.007G082200 | *-* | AD | GCCAGGTTATTCCTACTCAACTCAA | AACATCAAGAGCCAACCCCAT |
|  |  | exon | ACGGAAATGGGGAAACCACA | TGGGAGAGTTCCATGAGAGGA |
| Gorai.005G214900 | *-* | ES | CTTCTCACCATTCACTGCTTTCG | GAGAAATTCCAAGGGATCTGCCA |
|  |  | exon | TCTTGGAATCCATCCGAGCG | AAGGCCAACGGAACTGACAA |
| Gorai.011G087200 | *GrPHT3.1* | IR | ATCATTGCTTGGGAACTTGTACT | TACACTTCGCTGCACTTAGCA |
|  |  | exon | TATTCCCACTCCCAAGGAGCA | GCAGGATGCGAAACAATAGCA |
| Gorai.002G165700 | *-* | IR | AGTACTACCTGTTGATGATGGAT | TGCAGGCATAAAAGGTTCCA |
|  |  | exon | AACTTGTGGTTTTGCATGGC | ACAAAATGGAGTCCAAGGCA |
| Gorai.003G041300 | *GrHis3* |  | CGGTGGTGTGAAGAAGCCTCAT | AATTTCACGAACAAGCCTCTGGAA |
